# Dissociable impacts of perceived race and ascribed status in event-related brain potentials and multivariate network activity

**DOI:** 10.3758/s13415-026-01401-9

**Published:** 2026-02-05

**Authors:** Samuel A. Venezia, Eric D. Splan, Samwell Cleary, Jasmin Cloutier, Jennifer T. Kubota

**Affiliations:** 1 Department of Psychological and Brain Sciences, University of Delaware, 105 The Green, Newark, DE 19716 USA; 2 Johnson and Johnson, Philadelphia, PA USA; 3 Department of Political Science and International Relations, University of Delaware, 18 Amstel Avenue, Newark, DE 19716 USA

**Keywords:** Status, Race, Impression formation, Social categorization, Evaluation, Neural networks, Event-related brain potentials

## Abstract

Humans rapidly and efficiently categorize others with limited information, forming split-second impressions. Prior EEG person perception research has often focused on social categories derived from perceptual cues. However, impressions are frequently based on knowledge of someone. Little research has examined how person knowledge (or the interaction between perceptual category cues and person knowledge) influences the temporal unfolding of person perception, thereby missing a common experience of everyday encounters in which individuals have access to both. Using EEG, this study (*n* = 29) examined evoked event-related brain potentials (ERPs) and functional neural network responses previously associated with changes in attention and evaluation when perceivers categorized faces based on perceived race (i.e., Black or White) or ascribed socioeconomic status (i.e., high or low). Our findings indicate dissociations between ERPs and functional network dynamics during impression formation. Specifically, in immediate response to a face, perceived race shaped ERPs often associated with attention (P200) and motivation/evaluation (P300). However, ascribed status influenced coordination of the neural networks underlying attention/executive functions and social cognition/evaluation throughout the categorization task, suggesting that participants attended to and evaluated status in a sustained manner. Therefore, while race perception influenced ERPs, status did not. This was the opposite for the network analyses. These findings indicate that perceptual information (perceived race) and person knowledge (ascribed status) can influence impression formation in distinct ways: one in an immediate, evoked manner, and the other through the sustained coordination of functional networks.

**Supplementary Information:**

The online version contains supplementary material available at 10.3758/s13415-026-01401-9.

## Introduction

Humans rapidly and spontaneously glean categorical social information about others, including perceived race (Brewer, 1988; Fiske & Neuberg, 1990; Ito & Kubota, 2021; Quinn & Macrae, 2005). However, in daily life, people can also have specific knowledge about others (e.g., biographical information, morality, social status), often obtained through prior experience. Although impressions can be readily formed solely from perceptual information (e.g., visual features of a face), the availability of person knowledge (i.e., knowledge about someone) is beneficial for individuated construal of others (Anzellotti & Young, 2020; Barth et al., 2020; Cloutier et al., 2005, 2012; Cloutier et al., 2011a, 2011b; Cloutier & Gyurovski, 2013, 2014; Cohen, 1981; Dang et al., 2019; Fiske & Neuberg, 1990; Gyurovski et al., 2018; Kubota et al., 2023; Macrae et al., 2005; Mason et al., 2006; Mattan et al., 2019; Quinn & Macrae, 2005). It has been postulated that different social cognitive processes (e.g., categorization versus individuation) may be involved when forming impressions based on perceptual cues versus person knowledge (Anzellotti & Young, 2020; Bach & Schenke, 2017; Cloutier et al., 2011b; Todorov et al., 2007; Wagner et al., 2012). Existing work suggests that person knowledge may at times take precedence in the overall impression (Mattan et al., 2019). As such, examining impressions of others who vary in perceptual cues and person knowledge may provide a deeper understanding of how these two sources of information simultaneously impact person perception.

While person knowledge is vital for person-specific impressions, the majority of neuroscientific investigations of person perception have focused on impressions based solely on perceptual information (Chiao et al., 2008; Cloutier et al., 2011b, 2014; Freeman et al., 2009; Haaker et al., 2016; Hess et al., 2000; Mitchell et al., 2006). This stands in contrast to how existing models of impression formation construe real-world person perception, in which perceivers encounter targets who vary across multiple social categories and possess unique person knowledge (Brewer, 1988; Fiske & Neuberg, 1990). To examine how perceptual information and person knowledge influence the temporal unfolding of impressions, we investigated how perceived race (Black versus White individuals), ascribed social status (high or low socioeconomic status [SES]), and task affect the attentional and evaluative aspects of person perception. We assessed both the temporal unfolding of person perception with event-related brain potentials (ERPs) related to selective attention (P200) and motivation/evaluation (P300) (with confirmatory pre-registered hypotheses) as well as functional connectivity between regions in predefined attention/executive control and social cognitive/evaluative networks (with exploratory pre-registered analyses).

### Relevance of ERPs to perceptual information and person knowledge-based person perception

Event-related brain potentials are derived from the synchronous, summated postsynaptic firing of neurons in response to an event and have excellent temporal resolution (Fabiani et al., 2000). Event-related brain potentials are often named for their polarity and the approximate time that they occur (e.g., P200 is a positive-going component usually occurring at parietal sites around 180 ms after viewing a face). To investigate how perceptual information and person knowledge influence rapid and evoked attentional and evaluative processes, we examined two ERP components often implicated in person perception and categorization: the P200 and P300 components (Bartholow & Dickter, 2007; Ito & Bartholow, 2009; Ito & Kubota, 2014).

### Race perception and ERPs

Numerous studies find that humans process perceived race rapidly and efficiently, with the most reliable difference occurring at the P200 within 150–250 ms of viewing a face (Correll et al., 2006; Dickter & Bartholow, 2007; Ito & Urland, 2003, 2005; Ito et al., 2004; Kubota & Ito, 2007, 2017; Pesciarelli et al., 2021; Willadsen-Jensen & Ito, 2006, 2008). Previous work has implicated the P200 in selective attention and salience processing (Kubota & Ito, 2017). For example, greater P200 amplitudes have been observed among White perceivers when viewing perceived racial outgroups (i.e., Black faces or Asian faces) compared with perceived White faces (Kubota & Ito, 2007) or racially ambiguous faces (Willadsen-Jensen & Ito, 2006). Greater P200 responses to outgroup faces have also been found regardless of perceiver race when attending to faces of different perceived racial groups (Dickter & Bartholow, 2007; Volpert-Esmond & Bartholow, 2019; Willadsen-Jensen & Ito, 2008). Some outgroup members may be more strongly associated with threat (for non-ERP papers discussing the unconfounding of ingroup/outgroup and threat see Esses et al., 1993; Hong et al., 2024; Hong & Freeman, 2024; Kubota & Ito, 2014, 2015; Ito & Kubota, 2024), leading to greater P200 responses relative to ingroup members, but that does not imply that the P200 is sensitive to ingroup/outgroup distinctions (Volpert-Esmond & Bartholow, 2019, 2021). Therefore, although the P200 is often greater for salient outgroup members, there is ample evidence that the P200 is not simply tracking ingroup/outgroup distinctions, but rather the salience or threat of stimuli.

The P200 has been shown to track greater attention to perceived threatening or salient stimuli (e.g., greater attention to the most threatening outgroups, rather than any outgroup; see Ito & Kubota, 2021; Kubota & Ito, 2016 for discussion). For example, several investigations examining race processing using samples of self-identified East Asian participants found that, while P200 amplitudes were enhanced toward perceived White outgroup members, they were largest when participants viewed perceived Black outgroup members (Zhang et al., 2023; Zhou et al., 2020). Moreover, other investigations have found greater P200 amplitudes in response to perceived Black faces relative to perceived White faces among Black perceivers in the United States (Volpert-Esmond & Bartholow, 2019, 2021). These findings suggest the P200 may index greater selective attention to faces perceived as salient or those that convey evaluatively salient information (e.g., anger Kubota & Ito, 2007; Sheng & Han, 2012).

The P300 is a positive-going ERP component that is maximal over parietal regions between 300 and 800 ms in response to stimulus presentation (sometimes referred to as the P3b or Late Positive Potential). Henceforth, we will refer to this component as the P300. The P300 has been implicated in a variety of processes (Polich, 2012; Verleger, 1988), including sensitivity to stimulus novelty or frequency (Donchin & Coles, 1988; Friedman et al., 2001; Squires et al., 1975), memory and encoding (Karis et al., 1984), specifically context updating in working memory (Donchin & Coles, 1988; Johnson Jr & Donchin, 1980; see Polich, 2007 for review), and evaluative saliency of stimuli (Bartholow et al., 2001; Crites et al., 1995; Ito & Cacioppo, 2000; Ito et al., 1998; Kubota & Ito, 2007). Evaluative salience has taken the form of greater P300s to both negative and positive stimuli, including greater P300s to aversive or negative stimuli (Broyd et al., 2012; Delplanque et al., 2005; Foti et al., 2009; Hajcak & Olvet, 2008; Huang & Luo, 2006; Ito et al., 1998), as well as rewarding stimuli (Donchin, 1981; Glazer et al., 2018; Goldstein et al., 2006; Hughes et al., 2013; Polich, 2007; Sutton et al., 1965). Given the variety of processes indexed by the P300, researchers have posited that many of these findings have in common a sensitivity to motivationally salient stimuli (Crites et al., 1995; Crites & Cacioppo, 1996; Glazer et al., 2018; Ito & Bartholow, 2009; Ito & Kubota, 2021; Kubota & Ito, 2016; Nieuwenhuis et al., 2005; Pornpattananangkul et al., 2017; Weinberg & Hajcak, 2010). Because different social categories may be more or less motivationally salient during impression formation, researchers have examined the P300 in the context of person perception (Dickter & Bartholow, 2007; Ito & Bartholow, 2009; Ito & Kubota, 2021; Ito & Urland, 2003, 2005; Ito et al., 2004; Kubota & Ito, 2007, 2009; Willadsen-Jensen & Ito, 2006).

The P300 is sensitive to social categories motivationally salient to perceivers (Correll et al., 2006; Duval et al., 2013; Eimer & Holmes, 2002; Holmes et al., 2003; Ito & Cacioppo, 2000; Kubota & Ito, 2007; Kutas et al., 1977; Nieuwenhuis et al., 2005; Schupp et al., 2003, 2004). For example, among White U.S. participants, P300s were greater to perceived Black faces relative to perceived White or East Asian faces (Bartholow et al., 2006; Correll et al., 2006; Ito & Urland, 2003, 2005; Kubota & Ito, 2007; Willadsen-Jensen & Ito, 2006). Whether an ingroup or an outgroup member becomes socially salient is context-dependent (Bartholow & Dickter, 2007). In the U.S., Black individuals may be salient to White perceivers due to stereotypical associations of Black individuals with violence and danger (Kubota & Ito, 2014). Recent work on person perception has characterized the P300 as indexing a more deliberate evaluation of faces (e.g., reflecting on a categorization decision), rather than being directly implicated in attention or categorization (Amodio & Cikara, 2021).

### Status perception and ERPs

While a large body of EEG research has explored how perceived race influences person perception, limited studies have investigated how social status knowledge influences person perception with EEG (Breton et al., 2014, 2019; Chiao et al., 2008; Mattan et al., 2017; Pineda et al., 1994; Santamaría-García et al., 2015). Status (including SES) can be conveyed via knowledge (Mattan & Kubota, 2020; Mattan et al., 2017, 2018c). Existing research has found that both humans and nonhuman primates pay greater attention to high-status individuals (Deaner et al., 2005; Ly et al., 2011; Pavan et al., 2011) and that they receive more positive evaluations than low-status individuals (Anderson & Kilduff, 2009; Varnum, 2013). When status is conveyed through person knowledge, the ventromedial prefrontal cortex (VMPFC), a region thought to be involved in the generation of affective meaning and positive social evaluation (Cloutier & Gyurovski, 2014; Cloutier et al., 2012; Mattan et al., 2018a), has been found to respond to high-status others preferentially (Barth et al., 2020; Cloutier & Gyurovski, 2014).

However, evaluative responses depend on the social dimension conveying status (Mattan et al., 2017). For instance, neuroimaging research examining VMPFC responses has found that individuals with high financial status are evaluated more negatively (i.e., lower VMPFC response) than individuals with low financial status. In contrast, individuals with high moral status are evaluated more positively (eliciting a greater VMPFC response) than those with low financial status (Cloutier & Gyurovski, 2014; Gyurovski et al., 2018). Previous ERP research exploring status-based person evaluations, as indexed by P300 responses while participants categorized faces by status level (high versus low) and status dimension (moral versus financial), mirrored the VMPFC findings (Cloutier & Gyurovski, 2014). Gyurovski & colleagues (2018) found that status level and status dimension interacted to affect P300 amplitudes, such that P300s were greater for individuals with high financial and low moral status than for those with low financial and high moral status (Gyurovski et al., 2018). Recall that greater P300s have been associated with motivationally salient stimuli, particularly negative evaluative stimuli (Ito & Kubota, 2024). Therefore, although individuals, on average, attribute more positive evaluations to high-status targets, this may depend on numerous factors, including the social dimension that conveys status (Mattan et al., 2017). In this investigation, we focused on SES as a dimension of status, because perceived race and SES are often intertwined in the U.S. (although not perfectly correlated), and SES is conferred through knowledge, encompassing someone’s education, income, and occupation.

### ERP hypotheses

Based on previous ERP research, we predicted that P200s would differ as a function of perceived race, replicating prior findings (i.e., Confirmatory Prediction 1: greater P200s to perceived Black faces; Kubota & Ito, 2007). However, because less ERP research has examined how status influences P200s during categorization, and none, to our knowledge, has examined responses to others varying in SES, it was less clear how status may modulate race processing at the P200.

In contrast, in non-oddball paradigms, the P300 has been suggested to be sensitive to motivationally salient/relevant stimuli. However, the motivational salience of ingroup or outgroup members, in this case perceived White and Black faces, may depend on the context (Ito & Urland, 2005; Serafini et al., 2024). Because knowledge of others’ status may change the evaluative context, we predicted that perceived race and ascribed status would interactively influence P300s, with greater responses to the more motivationally salient social category. Specifically, existing ERP research has linked the P300 with participants’ negative biases towards perceived outgroup members (Black) faces compared with perceived ingroup members (White) faces (Dickter & Bartholow, 2007; Ito & Urland, 2003, 2005). Additionally, previous ERP research has linked the P300 with participants’ negative biases toward perceived negative status categories (e.g., low moral status) (Gyurovski et al., 2018). Therefore, we predict that race and status would interact to impact P300 amplitudes, such that P300s would be largest to Black low-status faces (stereotypically negative target) and smallest to White high-status faces (stereotypically positive target) (i.e., Confirmatory Prediction 2: greatest P300s to perceived Black low-status faces).

### Perceptual and person knowledge-based neural network modularity

In addition to ERPs, we examined how neural network coordination shaped person perception across the task. Recent work using EEG network approaches has demonstrated that functional connectivity can capture how distributed neural systems coordinate to support complex social-cognitive processes, including person perception (Forbes et al., 2018). Functional connectivity analyses assess statistical dependencies among neural signals over time, indexing how brain regions coordinate during rest or task engagement. In contrast, ERP analyses characterize time-locked changes in electrical activity evoked by discrete events. Thus, ERPs and functional connectivity offer complementary insights into psychological processes. Event-related brain potentials provide fine-grained temporal resolution, allowing researchers to identify when specific cognitive operations occur following stimulus onset. In contrast, functional connectivity adopts a systems-level perspective, characterizing how multiple brain regions interact and integrate over extended periods of task performance. This approach can reveal patterns of interdependence among neural systems that are not readily captured by univariate, event-locked analyses.

Importantly, functional connectivity can index intrinsic and task-dependent network organization, providing insight into psychological processes such as attention allocation, evaluative processing, and default mode engagement, which may unfold over longer timescales than those typically isolated by ERPs. Network analyses further enable the investigation of simultaneous interactions across multiple functional systems, reflecting the integrated nature of person perception. Event-related brain potentials analyses characterize the temporal dynamics and magnitude of event-locked neural responses, whereas EEG functional connectivity analyses characterize task-dependent coordination among distributed neural systems. Whereas ERPs capture moment-to-moment evoked responses (e.g., P200, P300), functional connectivity characterizes task-dependent coordination among distributed neural systems (attention/executive function and social cognition/evaluation).

Consistent with prior work demonstrating the utility of multivariate network approaches in person perception (Forbes et al., 2018; Mattan et al., 2018b), we leveraged these complementary methods to examine how perceived race, ascribed social status, and task shape both evoked neural responses and broader patterns of network coordination over time. Within the person perception literature, employing multivariate network approaches has helped detect patterns of neural activity through a data-driven method that conventional univariate analyses (e.g., ERPs) are unable to detect (Mattan et al., 2018b), such as activity in networks involved in attention, memory, and executive function (Forbes et al., 2018), as well as social cognitive and evaluative processes (Barth et al., 2020; Cloutier et al., 2017; Handley et al., 2021; Mattan et al., 2018b). Therefore, examining functional connectivity among networks previously implicated in person perception, specifically those involved in processing perceived race and ascribed status, could provide further insights, in combination with ERP analyses, into how the combination of these social dimensions influences the coordination of person-perception relevant networks during categorization over time.

Considering previous work suggesting that social status may not be processed as rapidly as perceived race (Gyurovski et al., 2018), these complementary network analyses help further outline the relationship, or lack thereof, between these social dimensions. A theoretical advantage of functional neural network analyses is that they enable researchers to use dynamic functional network characterizations and compare their outcomes with existing person perception theory, thereby helping to generate new hypotheses (McGrath et al., 2024). For example, finding that person knowledge dominates evaluative- and attention-based network coordination during categorization may suggest that person knowledge is a dominant component of impression formation over perceptual information.

Connectivity among nodes within predefined functional networks implicated in attention/executive control and social cognition/evaluation was examined across task conditions (van Straaten & Stam, 2013). Nodes within either network were derived from previous fMRI literature related to impression formation of others varying in perceived race and ascribed status (Mattan et al., 2017, 2018a, 2018b, 2018c) to investigate the degree of their interconnection and effective communication when categorizing faces (Newman, 2004; Rubinov & Sporns, 2010; Sporns, 2011). As such, we opted to construct two networks of brain regions: one believed to support attention and executive control broadly, and the other to support social cognition and evaluation during impression formation. Their relevance to social cognition as well as their potential relevance to attentional (in this case, P200) and evaluative/motivational (P300) ERP responses drove this choice.

### Attention/Executive function neural network

To examine differences in network coordination related to attention and executive function, we focused on a core set of regions including the inferior parietal sulcus (IPS), dorsolateral prefrontal cortex (DLPFC), ventrolateral prefrontal cortex (VLPFC), and dorsal anterior cingulate cortex (DACC). With respect to attention, the IPS is a key region supporting selective attention (Gillebert et al., 2011; Vandenberghe et al., 2005, 2012), integrating sensory information with motor planning to facilitate attentional focus on spatial locations (Coull & Frith, 1998). Within person perception, the IPS has been implicated in status-based attention, particularly during judgments of relative social distance and rank (Chiao et al., 2009; Koski et al., 2015). Prior work demonstrates greater IPS activity when attending to targets closer in relative status (Cloutier & Gyurovski, 2013; Zink et al., 2008), suggesting a role for this region in the attentional demands of social status comparisons (Mattan et al., 2017; Qu et al., 2017).

Executive functions are also critical for navigating complex social environments, including intergroup interactions, by supporting response selection, conflict monitoring, and memory updating (Macrae et al., 1999; Pearson et al., 2013). Although executive control relies on a distributed network, the DLPFC, VLPFC, and DACC are consistently implicated in social cognition. Prominent theories of executive function propose that the ACC detects conflict during response processing, while regions, such as the DLPFC, implement control adjustments to optimize performance (Carter & van Veen, 2007; Carter et al., 1998; Egner & Hirsch, 2005; MacDonald et al., 2000; Mansouri et al., 2009; Miller & Cohen, 2001; Ridderinkhof et al., 2004; Roberts & Hall, 2008). In line with this framework, both the ACC and DLPFC have been linked to race- and status-based impression formation (Kubota et al., 2012; Mattan et al., 2017, 2018c). The DLPFC shows heightened responses to individuals perceived as dominant (Marsh et al., 2009) and during interactions involving conflict with similarly dominant partners (Haaker et al., 2016). Converging evidence further suggests that the DLPFC operates in concert with the ACC to regulate biased responses, supporting alignment between behavior and egalitarian goals (Amodio, 2014; Amodio et al., 2006, 2008; Ito & Bartholow, 2009; Kubota et al., 2012; Mattan et al., 2018c). The VLPFC is commonly associated with the regulation of affect and inhibitory control, particularly in social contexts involving negative emotion (Hartley & Phelps, 2010; Ochsner et al., 2012; Tupak et al., 2014; Wager et al., 2008). Although its role in regulating intergroup bias has received less direct attention (Telzer et al., 2015), evidence suggests that the VLPFC may support response monitoring and behavioral inhibition during status-based evaluations, especially when interacting with highly dominant or high-status individuals (Marsh et al., 2009). Together, these findings motivated the inclusion of the IPS, DLPFC, DACC, and VLPFC as predefined nodes in the attention/executive function network.

### Social cognition/Evaluation neural network

To assess differences in social cognition and evaluative network coordination, analyses focused on the ventromedial prefrontal cortex (VMPFC), insula, dorsomedial prefrontal cortex (DMPFC), and superior temporal sulcus (STS)/temporoparietal junction (TPJ). Extensive prior research has linked both the VMPFC and insula to person evaluation. The VMPFC is engaged during social evaluative processes, including impression formation and explicit or implicit evaluation when person knowledge is available (Dang et al., 2019; Delgado et al., 2016; Roy et al., 2012). Meta-analytic and review work suggests that the VMPFC supports the flexible integration of affective and evaluative information from multiple sources, facilitating the construction of subjective value and meaning (Delgado et al., 2016; Roy et al., 2012) by integrating evaluation-relevant information from various sources (Dang et al., 2019; Mattan et al., 2017). Consistent with this role, VMPFC activity is sensitive to social evaluations involving person knowledge, including knowledge about others’ social status (Barth et al., 2020; Cloutier & Gyurovski, 2014; Cloutier et al., 2012; Mattan et al., 2018a). The insula likewise contributes to evaluative processing, particularly in response to salient, affectively charged, or socially relevant stimuli (Berntson et al., 2011). Prior studies report increased insula activity to pathogenic cues (Calder et al., 2007; Heining et al., 2003), moral transgressions (Borg et al., 2008; Ying et al., 2018), and during categorization of salient person knowledge (e.g., political orientation; Cikara et al., 2017). Of relevance, the insula has been implicated in intergroup evaluation, including responses to race and status cues (Cloutier & Gyurovski, 2014; Cloutier et al., 2017; Mattan et al., 2017, 2018c; Skinner & Hudac, 2017).

Beyond evaluation, effective social interaction requires inferences about others’ mental states. Regions, such as the DMPFC and STS/TPJ, are central to mentalizing processes during impression formation (Cloutier et al., 2008; Dang et al., 2022; Deen et al., 2015; Fletcher, 1995; Handley et al., 2021; Hoffman & Haxby, 2000; Mitchell et al., 2005; Schurz et al., 2014; Vogeley et al., 2001). The DMPFC reliably shows greater activation during mental state inferences, such as judgments about preferences or secondary emotions, relative to nonsocial judgments (Handley et al., 2022, 2023; Mitchell et al., 2005, 2006). Similarly, the TPJ is engaged when individuals reason about others’ beliefs and perspectives, compared with processing nonmentalistic social or physical information (Saxe & Wexler, 2005; Young et al., 2010). The STS, a region associated with social perception, is recruited during the interpretation of others’ intentions, gaze, and dynamic social cues (Allison et al., 2000; Deen et al., 2015; Pelphrey et al., 2003, 2004a, 2004b; Zilbovicius et al., 2006). Importantly, both the DMPFC and STS/TPJ have been shown to vary as a function of group membership and status, with reduced responses to outgroup targets (Bruneau et al., 2018; Firat et al., 2017; Handley et al., 2023; Harris & Fiske, 2006) and increased responses to high-status individuals (Mattan et al., 2018a, 2018b, 2018c). Collectively, these regions have been consistently implicated in race- and status-based social cognition and evaluation (Kubota et al., 2012; Mattan et al., 2018c). Accordingly, the predefined nodes in the social cognition/evaluation network included the VMPFC, insula, DMPFC, and STS/TPJ.

### The present study

This research offers a theoretically and methodologically significant advancement in the neuroscience of intergroup person perception. This research advances theory by integrating two cues commonly available during encounters, person knowledge and perceptual cues, which are relatively unexplored in combination in EEG/ERP research, to better understand when and how they shape person perception (e.g., interactively or additively). This helps refine existing models of person perception, which often rely on single cues (e.g., inferred race alone) or two perceptual cues (e.g., inferred race and gender). This research advances the methodology in the neuroscience of intergroup person perception by using ERPs and network analyses to examine both immediately evoked and sustained responses, thereby enhancing our understanding of how multiple social dimensions shape person perception in real time (both immediately and over time).

More specifically, although previous research has examined how perceptual cues or person knowledge affect impression formation, to our knowledge, no study has examined how perceptual cues and person knowledge simultaneously influence the temporal and spatial unfolding of impression formation. This question is particularly relevant to the social and cognitive psychology of impression formation because a significant portion of research relies solely on perceptual cues (e.g., inferring race or trustworthiness from a face), which can bias conclusions about how impression formation unfolds. In contrast, person knowledge tends to favor individuation and is available in many encounters. Examining the temporal unfolding of person perception based on various social cues and knowledge is essential because many models of impression formation posit the rapid, ordered unfolding of discrete psychological processes (Brewer, 1988; Bodenhausen et al., 1999; Fiske & Neuberg, 1990; Hugenberg & Bodenhausen, 2004; and ERP-specific, Amodio & Cikara, 2021; Amodio et al., 2014; Kubota & Ito, 2015). For example, several models of person perception based on ERP research suggest that perceptual cues are processed rapidly, with the influence of person knowledge occurring later (e.g., Kubota & Ito, 2015, for a review). However, recent theories have also suggested that the categorization process is more dynamic and not solely dictated by visual information but also by top-down factors (e.g., visual attention or goals) (Freeman & Ambady, 2011; Freeman et al., 2011; Volpert-Esmond et al., 2017). These accounts have indeed found support for dynamic and iterative person perception. However, the influence of person knowledge on person perception of visual social cues has not been examined, leaving open the question of whether the processing of person knowledge and perceptual cues influences person perception dynamically (interactively) or in an additive fashion over time.

To examine how perceived race, ascribed status, and task influence both ERPs and the coordination of functional neural networks associated with attention-executive function and social cognition-evaluation, the present investigation tasked participants with viewing faces that varied in perceived race and ascribed social status while rapidly categorizing them by either dimension. For all analyses, we tested the interaction among perceived race, ascribed social status, and categorization task. Our pre-registered, confirmatory hypotheses focused on ERP amplitudes in the P200 (i.e., Confirmatory Prediction 1: greater P200s to perceived Black faces [main effect of perceived race], e.g., Kubota & Ito, 2007) and P300 (i.e., Confirmatory Prediction 2: greatest P300s to perceived Black low-status faces [interaction among race and status]), complemented by functional neural network hypotheses (Exploratory Analysis: examining functional connectivity between regions in predefined attention/executive control and social cognitive/evaluative networks as a function of perceived race, ascribed status, and task (Venezia et al., 2024).

## Methods

### Participants

Forty-two self-identified White U.S. participants (31 women, 9 men, 1 nonbinary, 1 did not report), non-Hispanic, right-handed neurotypical participants between the ages of 18 to 24 (*M*_*age*_ = 18.87 years) were recruited for this study through the University of Delaware’s SONA system (*n* = 36) or were paid volunteers from the local community (*n* = 6). Participants were compensated at a rate of 1 study credit or $10 per hour, for a total of 2.5 study credits or $25. Participants were required to have lived in the U.S. for at least 5 years before participating in the study to ensure baseline exposure to U.S. racial stereotypes.

Exclusions were based on our pre-registration (Venezia et al., 2024) (https://osf.io/gytj5). Participants were excluded from participation if they reported a severe head injury or abnormal neurological history. Four participants were excluded for technical issues that occurred during their sessions. One participant was excluded for failing to follow study protocols. One additional participant was excluded due to poor signal quality at critical electrode sites and excessive alpha activity in their waveform. A further seven participants were removed for having fewer than our a priori threshold of 75 usable trials per condition, resulting in a final sample of 29 usable participants (21 women; 7 men; 1 nonbinary) between the ages of 18 to 24 (*M*_*age*_ = 18.97 years). Participants were given informed consent following the University of Delaware’s independent review board guidelines.

### Sample size

When the study was first pre-registered in 2018, we planned to examine how perceived race, ascribed status, categorization task, and the individual difference of external motivation to control racial prejudice (Plant & Devine, 1998) shaped P200 and P300 amplitudes. Due to the individual difference measure, the original sample size was 135 participants (see OSF pre-registration: https://osf.io/m23sb; Status-Race EEG Pre-reg Amendment 5). However, data collection did not start until Fall 2019 and was interrupted by COVID. Therefore, before data analysis, we submitted a pre-registered amendment clarifying that we would not examine individual differences and that we were powered at 29 participants to explore the interaction between perceived race, ascribed status, and the categorization task. Moreover, we conducted a post hoc sensitivity analysis to ensure that our observed effects were adequately powered (see Supplemental Materials S1). OSF pre-registration https://osf.io/gytj5 (Venezia et al., 2024) is the final pre-registration for the experiment (see Supplemental Materials S8 Pre-registration Amendments). Therefore, the final sample size is 29 participants.

To calculate power, we conducted a power analysis using the PANGEA package (Westfall, 2015) to identify the sample size. As this was the first study, to our knowledge, to examine how perceived race and ascribed status impacted ERPs, the variance and effect size parameters were not a priori predictable. Therefore, we used default variance parameters in PANGEA (var[error] =.2; var(P*E) = 0.04) to estimate power for a 2 (Ascribed Status: high, low) × 2 (Perceived Race: Black, White) × 2 (Categorization Task: Race, Status) within-participants design. Results suggested that 29 participants would be sufficiently powered to detect a significant interaction among Status*Race*Task at an effect size as small as *d* = 0.15, 1–*β* = 0.80. Although we did not have explicit P200 and P300 hypotheses regarding the task, P300s are modulated by task goals (see Amodio & Cikara, 2021, for a discussion). Therefore, the study was powered to detect the three-way interaction.

### Stimuli

We used 60 photos of male faces (30 perceived Black, 30 perceived White) from two existing databases (Ma et al., 2015; Meissner et al., 2005). These stimuli were previously used in other neuroimaging studies and equated on perceived race, emotional expression, trustworthiness, dominance, attractiveness, and likability (Mattan et al., 2018a, 2018b). Additionally, the images were also equated on contrast and luminance using the SHINE toolbox (Willenbockel et al., 2010). Each face was presented twice per block, for a total of 16 presentations per face. Faces were presented in red or blue shirts, counterbalanced across participants.

### Procedure

#### EEG session

After obtaining written consent and undergoing EEG cap and electrode application, participants completed a brief introduction to the color-status pairings. Then, they completed two practice categorization blocks, categorizing faces by perceived race or social status. Each practice block consisted of eight trials. After the practice blocks, participants verified that they understood the task and completed eight blocks of the categorization task. Within the main task, blocks consisted of 120 trials in total. Four blocks tasked participants with categorizing faces by perceived race, and the other four by ascribed social status. Participants used the one or two keys on a button box with their right-hand middle and index fingers to respond. The response box was on a stand next to the participant’s dominant (right) arm. The task order alternated between each block and was counterbalanced across participants. Additionally, color-status associations, each face’s status level, and key mapping were counterbalanced across participants. The presentation of each face was randomized for each participant. At the end of the categorization task, participants’ EEG cap was removed. Then, they completed a status recall task, an evaluative priming task (Mattan et al., 2019), and a demographics questionnaire (see Supplemental Materials S2) and were debriefed and compensated.

#### Status training

Before starting the categorization task, each participant was first trained to associate colored shirts with either high or low SES. Before seeing which colored shirt represented either high or low SES, participants read the following definition of SES as defined by the APA (American Psychological Association, n.d.): *“Socioeconomic status is the social standing or class of an individual. It is often measured as a combination of education, income, and occupation. People may be either high or low in socioeconomic status.”​* Following the brief status definition, participants viewed silhouettes with either a red or a blue shirt. Accompanying the silhouette was text that ascribed the shirt color as being either high or low status.

### Categorization task

**Fig. 1:**
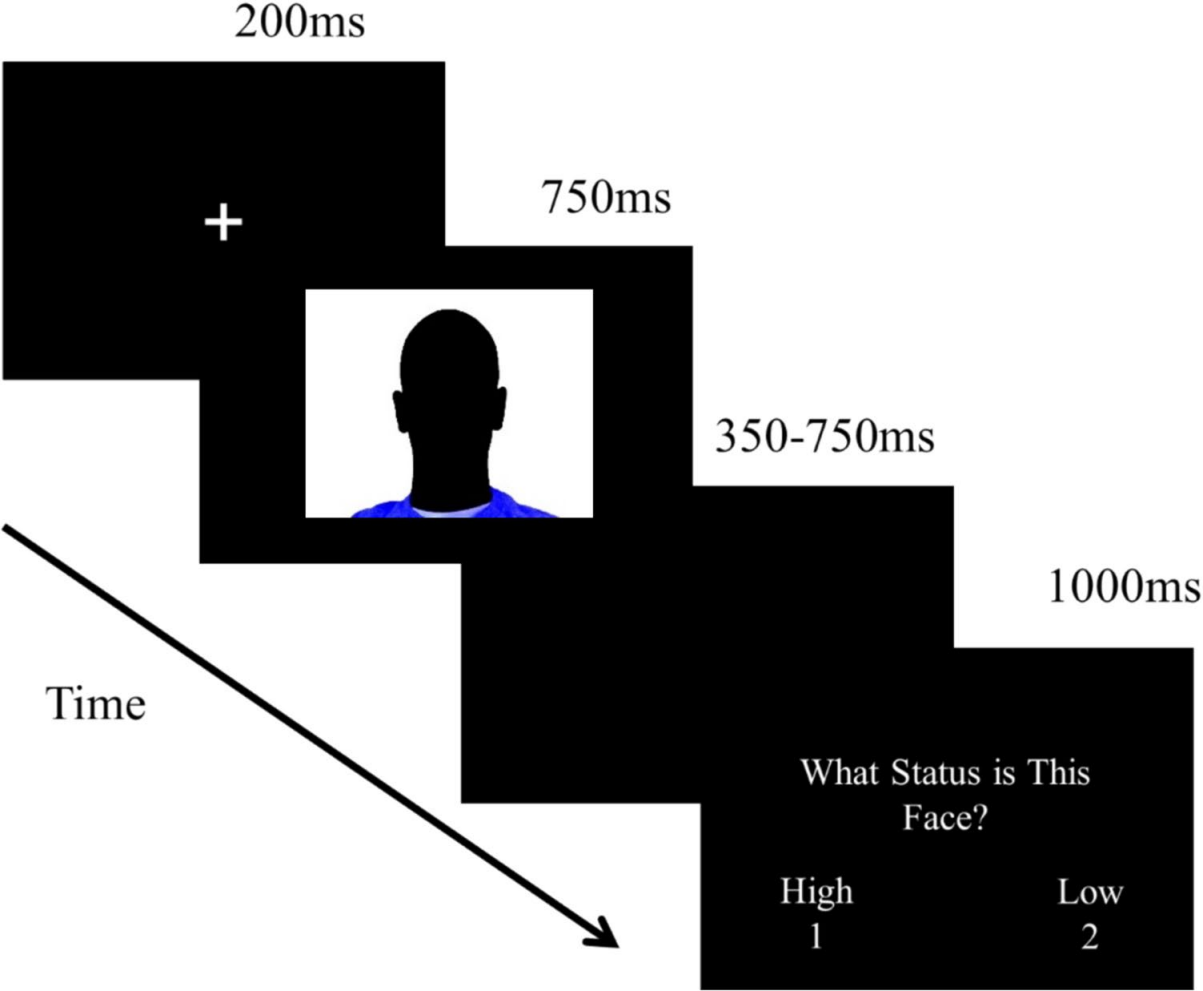
*Example of a trial within the categorization task*. Participants viewed a centrally presented fixation cross for 200 ms before each face was presented for 750 ms. Following face presentation, participants saw a jittered black screen before being prompted to categorize each face by either perceived race or ascribed status

Stimuli were presented using E-Prime 3.0 software. The first eight trials were practice using different faces sampled from the same four conditions as the main task. The presentation of eight practice trials was randomized. The main task consisted of 120 trials, for a total of 960 across the main categorization task. Face presentation was randomized within each block, and each face was presented twice per block. Each trial began with a centrally presented fixation cross for 200 ms, followed by a target face for 750 ms (Fig. 1). An interstimulus interval black screen was then presented for a jittered random interval between 350–750 ms. Following this screen, participants were prompted to categorize targets by either perceived race or social status (e.g., “What race/status is this face?). The two response options were labeled “1” or “2” on the computer monitor, which corresponded to either perceived race categorization (i.e., perceived Black or White face) or ascribed status categorization (high-status or low-status). The response options were counterbalanced across conditions to avoid a possible conditioned association of responses (e.g., White and high-status being associated with the same response key). Each trial terminated upon categorizing the face or 1,000 ms after the categorization prompt if participants did not respond within 1,000 ms. Following categorization, a second jittered blank screen (intertrial interval) was presented, varying randomly between 250 and 500 ms, before participants continued to the subsequent trial. Between blocks, participants were informed of the categorization task in the subsequent block (e.g., "Please take 1 min to rest, during this next set of trials, please categorize the individuals by their status.").

### Behavioral data analyses

We analyzed participants’ behavioral categorization data by examining their log-transformed response times of correct trials. Additionally, we analyzed participants’ accuracy rates for each condition (see Supplemental Materials S2.3). To probe response times, we fit log-transformed response times as a function of the three within-participant conditions. To investigate the effects of perceived race, ascribed status, and task on categorization speed, a 2 (Task Block: Status Categorizations, Race Categorizations) × 2 (Perceived Race: Black faces, White faces) × 2 (Ascribed Status: Low-Status, High-Status), linear multilevel mixed-effects regression was conducted in the R programming language (R Core Team, 2017) using the LMER function. Random effects were determined using the BUILDMER package in R (Voeten, 2021). To determine optimal model fit for all multilevel regression models reported (including ERP and functional connectivity analyses), we used a procedure identical to the one used in Mattan et al. (2019) that was adapted from existing research for determining random effects structures (Bates et al., 2015a, 2015b; see Supplemental Materials S3). We modeled a random intercept for each participant and, when possible, random slopes for each predictor, ensuring the number of random slopes was maximal without overfitting the model (see Supplemental Materials S3). Finally, in cases of significant interactions, we conducted follow-up models to test for simple effects.

### EEG recording and ERP processing

The EEG was recorded in a nonshielded room using a 128-channel Biosemi ActivTwo recording system at a sampling rate of 2048 Hz. Impedances were below 5 kΩ for recording. A horizontal electrooculogram (EOG) was recorded by electrodes placed on the right and left of both eyes. Vertical EOG was recorded through two electrodes above and below the participant’s left eye. Before signal cleaning, the EEG and EOG signals were referenced to the participant’s scalp average. The EEG signal cleaning and analysis were conducted in MATLAB using EEGLAB and the ERPLab toolbox. The EEG signal was processed using best practices (Luck, 2014). We first ran an IR Butterworth high-pass filter with a cutoff of 0.1 Hz and a low-pass filter of 30 Hz. A 60-Hz notch filter followed this before downsampling the EEG data to 512 Hz. Break periods of 3 s or longer were then removed. Bad channels were then identified visually and interpolated using spherical interpolation. An independent component analysis (ICA) was then run to identify and remove eyeblink components using the ICABlinkMetrics function (Pontifex et al., 2017) in EEGLab. Additionally, the ICLabel function (Pion-Tonachini et al., 2019) identified any remaining components associated with muscle, eye, ECG, or noise artifacts, using a 90% certainty threshold.

Epochs of interest were created with a 200 ms baseline, extending 1,000 ms poststimulus onset, and were binned by the respective condition. Following epoch creation, we computed trial-by-trial interpolation on each created epoch to maximize the total usable trials (Ben-Shachar, 2018). To remove remaining epochs with excessive noise, epochs were then submitted to a moving window threshold, which rejected epochs that showed a variation of signals greater than 100 microvolts within a 200-ms window, and then to a simple voltage threshold, which excluded epochs that contained EEG signal that was either less than − 75 μV or exceeded 75 μV. Participants with fewer than 75 trials in any condition (*n* = 7) were excluded at this point. After removing epochs with excessive noise, the remaining participants’ epochs were averaged for each condition to create ERPs.

To determine maximal scalp location, each component was assessed over a frontal (Fz international 10–20 system; C21 Biosemi scalp site), central (Cz international 10–20 system; A1 Biosemi scalp site), and parietal electrode (Poz international 10–20 system; A19 Biosemi scalp site), along the scalp midline. Time windows for the P200 and P300 analyses were determined a priori based on prior research and are reported in the pre-registration (Venezia et al., 2024; Kubota & Ito, 2007; 2016). Visual inspection of the grand-average waveforms confirmed the scalp location but not the analysis time window around the peak amplitude (as this was pre-registered).

We anticipated that the P200 epoch window would be 100 ms in length, centered on the peak, and that the P300 window would be 300 ms in length, also centered on the peak. After data collection, the P200 was quantified as the maximal positive amplitude between 150–250 ms post-face onset. The P300 was quantified as the average positive voltage between 270–570 ms post-face onset. Although the P200 and P300 were pre-registered as the largest at parietal locations, the exact electrode location was not pre-registered. To verify scalp locations for ERP analyses, the three electrode sites were regressed on amplitude before condition-based analysis. Results revealed that the P200 was greater at the central (Cz) relative to the frontal (Fz) site, *b* = 1.205, *SE* = 0.276, *CI*_*95%*_ = [0.663, 1.744], *t*(665) = 4.366, *p* < 0.001. Additionally, consistent with the pre-registration, maximal at the parietal site (Poz) relative to either Cz, *b* = 9.338, *SE* = 0.276, *CI*_*95%*_ = [8.798, 9.878], *t*(665) = 33.873, *p* < 0.0001, or Fz, *b* = 10.541, *SE* = 0.276, *CI*_*95%*_ = [10.001, 11.082], *t*(665) = 38.239, *p* < 0.0001. The P300 was larger at Cz relative to Fz, *b* = 2.202, *SE* = 0.218, *CI*_*95%*_ = [1.774, 2.630], *t*(665) = 10.084, *p* < 0.001. Additionally, consistent with the pre-registration, the P300 was maximal at Poz relative to Cz, *b* = 7.923, *SE* = 0.218, *CI*_*95%*_ = [7.495, 8.351], *t*(665) = 36.282, *p* < 0.001, or Fz, *b* = 10.125, *SE* = 0.218, *CI*_*95%*_ = [9.697, 10.553], *t*(665) = 46.367, *p* < 0.001. As such, for our final analyses, we examined P200s and P300s at Poz.

To probe the effects of perceived race and status on ERPs, a 2 (Task Block: Status Categorizations, Race Categorizations) × 2 (Perceived Race: Black faces, White faces) × 2 (Ascribed Status: Low-Status, High-Status) linear multilevel mixed-effects regression was conducted. Amplitude was the DV. To account for participant-level variation in ERP amplitudes, we modeled random intercepts for each participant and random slopes for the predictors, ensuring the number of random slopes was maximal without overfitting the model (see Supplemental Materials S3). Finally, in cases of significant interactions, we conducted follow-up models to test for simple effects.

#### Functional connectivity network modularity analyses

Next, we describe the data analysis procedures for the functional connectivity analyses. The brain is organized into multiple interacting functional subsystems, or modules, that are relatively specialized yet dynamically coupled to support cognition and behavior (Bassett et al., 2010; Simon, 1962). Network neuroscience approaches provide tools for characterizing how patterns of functional connectivity are organized within and between subsystems. We used functional network connectivity analyses to examine the extent to which neural systems supporting attention/executive function and social cognition/evaluation were differentially coordinated during categorization of perceived race and ascribed social status across task conditions and time. These analyses were conducted as pre-registered exploratory analyses using network-based functional connectivity methods (Rubinov et al., 2009). Functional neural networks refer to sets of brain regions that tend to exhibit coordinated activity and are thought to support related cognitive processes (e.g., attention, memory). At rest, functional connectivity is organized into a modular architecture, including well-characterized large-scale networks such as the default mode network (Raichle, 2015). During task performance, this modular organization can be reconfigured, reflecting changes in the balance between within-network segregation and between-network integration in response to task demands (Félix & Wagner, 2008). Individual differences in resting-state network modularity have been associated with variability in cognitive performance across tasks (Stanley et al., 2014; Stevens et al., 2012), motivating the use of modularity-based approaches to examine task-related changes in network organization.

Modularity analyses were conducted on a priori regions belonging to attention/executive function and social cognition/evaluation networks, defined based on previous fMRI research on perceived race and ascribed social status processing (Mattan et al., 2017, 2018a, 2018b, 2018c). The EEG data used for the modularity analyses were preprocessed by using the same pipeline as the ERP analyses, with the exception that the low-pass filter was set to 55 Hz to accommodate the alpha (8–15 Hz), beta (15–30 Hz), and gamma (30–55 Hz) frequency bands.

After re-preprocessing the EEG data, we conducted fixed-partition (select) network modularity analyses to compare connectivity within predefined networks of interest, specifically the attention/executive function and social cognition/evaluation networks, to between-network connectivity across the whole brain (Forbes et al., 2018). These analyses quantified the extent to which functional connectivity was preferentially organized within predefined networks compared with whole-brain connectivity. The EEG source localization was performed by using the MNE-Python toolbox (Gramfort et al., 2013, 2014). A forward model was computed by using a standard MNI template brain (Fischl, 2012), and source estimates were obtained by projecting scalp-recorded EEG data into template-based source space using dynamic statistical parametric mapping (dSPM), constrained to the cortical surface. The resulting source estimates were then parcellated using the Desikan–Killiany atlas (Desikan et al., 2006), an automated cortical labeling system that subdivides the cortex into 68 regions of interest. All regions of interest were cortical, and this atlas has been widely used in network-based analyses of EEG data (Bathelt et al., 2013; Wirsich et al., 2021). Following parcellation, time-series estimates for each of the 68 cortical nodes were extracted for every trial over the 1000 ms period following stimulus onset.

The MNE spectral connectivity analyses were then used to estimate coherence and phase-locking values (PLV; Lachaux et al., 1999) for each pair of nodes averaged across trials for each condition, yielding 68 × 68 connectivity matrices for three frequency bands. We selected frequencies based on a previous investigation that used a similar network-based analytical approach (Liu et al., 2021). As such, separate matrices were calculated for the alpha (8–15 Hz), beta (15–30 Hz), and gamma (30–55 Hz) wavebands. As the functional connectivity analyses were exploratory, we opted to select this range of frequency bands as connectivity across these frequencies may reflect processes important for social cognition, such as social evaluations (Mu, Fan, Mao, Han, 2008) working memory (Buzsaki, 2006; Forbes et al., 2018; Sauseng et al., 2005), goal-relevant response inhibition (Klimesch et al., 2011; Leitner et al., 2014), attention (D’Andrea et al., 2019; Klimesch, 2012; Rouhinen et al., 2013, 2020), integration of working memory (Baldauf & Desimone, 2014; Jensen et al., 2007; Rouhinen et al., 2013), and intergroup evaluations (Gutsell & Inzlicht, 2012; Han, 2018; Perry et al., 2010). Analyzing the three frequency bands offered insight into which neural process may be most implicated while integrating perceived race, ascribed status, and task during categorization.

Phase-locking values (PLVs) were first computed to construct functional connectivity matrices, which index the consistency of phase differences between pairs of ROI time series across specific frequency bands. The PLVs range from 0 to 1; higher values indicate greater phase synchrony between pairs of neural signals and provide a measure of pairwise functional coupling (Forbes et al., 2018). Modularity (Q_select_) was computed on weighted, PLV-based functional connectivity networks to quantify the extent to which connectivity was organized according to predefined network partitions, such that within-network synchrony exceeded that expected under a whole-brain null model. Higher modularity values indicate greater segregation of connectivity within the predefined networks than between networks. In contrast, values near zero or negative indicate no greater within-network organization than expected based on whole-brain connectivity. Modularity was computed using functions from the Brain Connectivity Toolbox (Rubinov & Sporns, 2010), separately for each predefined network, and these values were used to compare network-specific patterns of functional segregation across conditions. Select network modularity (Q_select_), defined as the modularity of predefined network partitions (Forbes et al., 2018), was computed as:

$${Q}_{select}= \frac{1}{2{m}}\sum_{{i},\;j}({{\rm A}}_{{i},\;j}- {P}_{{i},\;j})\delta ({g}_{i},{g}_{j})$$

where ${\mathrm{A}}_{{i},j}$ represents the PLV-based functional connectivity between nodes ${i}$ and $j$, ${m}= \frac{1}{2}{\sum }_{{i},j}{{\rm A}}_{{i},j}$ is the total connection weight in the network, ${P}_{{i},j}= \frac{{k}_{{i}}{k}_{ j} }{2{m}}$ denotes the expected connection weight under a strength-preserving null model (${k}_{{i}}$ denotes the strength (i.e., sum of PLV weights) of node ${i}$), and $\delta \left({g}_{i},{g}_{j}\right)=1$ when nodes ${i}$ and $j$ belong to the same predefined network, and 0 otherwise. Q_select_ denotes the modularity value computed for a predefined (fixed) network partition. Connectivity was examined within specific frequency bands, brain networks, and experimental conditions. ${\mathrm{A}}_{{i},\text{ j}}$ represents the observed edge weight between nodes $\mathfrak{i}$ and $\mathrm{j}$ defined as the phase-locking value (PLV), indexing connection strength in the EEG-derived functional connectivity network. ${\mathrm{P}}_{{i},\text{ j}}$ denotes the expected edge weight under a strength-preserving null model, which accounts for whole-brain connectivity while maintaining node strength distributions. Graph analyses were conducted on PLV values computed within three frequency bands from 0 to 1,000 ms poststimulus onset. For each subject, condition, and frequency band, a 68 × 68 weighted, undirected adjacency matrix was constructed, with nodes corresponding to cortical regions and edges weighted by PLV values. Before modularity analysis, adjacency matrices were proportionally thresholded to ensure a sparse network structure. Specifically, only the strongest 30% of connections were retained, whereas weaker connections were set to zero, resulting in sparse, weighted, undirected graphs (Liu et al., 2011, 2013). Modularity (Q_select_) was then computed on the full sparse graph using predefined network partitions corresponding to the attention/executive function and social cognition/evaluation networks. These modularity values were then used to examine condition-dependent differences in functional segregation within each frequency band and network.

Our nodes of interest were those previously identified as implicated in attentional/executive functions and social cognitive/evaluative processes (Mattan et al., 2017). Because the P200 and P300 have been postulated to be related to salience/selective attention and social cognition/evaluations, respectively, we initially aimed to mirror the ERP analyses by characterizing networks that reflected these two processes. Furthermore, because select modularity scores would be calculated across a full experimental trial, we wanted to explore whether we could detect differences in attention and evaluation that are not captured by ERP components. These connectivity analyses allowed us to examine the specific underlying processes in each condition across the entire trial rather than just within ERP time windows. Our analyses focused on differences in select modularity within each frequency band and across both networks, for each experimental condition. The attention/executive function network consisted of the IPS, VLPFC, DLPFC, and DACC. The social cognitive/evaluative network consisted of the VMPFC, DMPFC, insula, and STS. Select network modularity scores were entered into a 2 (Categorization Task: Race, Status) × 2 (Ascribed Status: High, Low) × 2 (Perceived Race: Black, White) repeated measures multilevel regression in the R programming language (R Core Team, 2017) using the LMER function. Random effects were again determined by using the BUILDMER package in R (Voeten, 2021).

## Results

### Behavioral response times

**Fig. 2:**
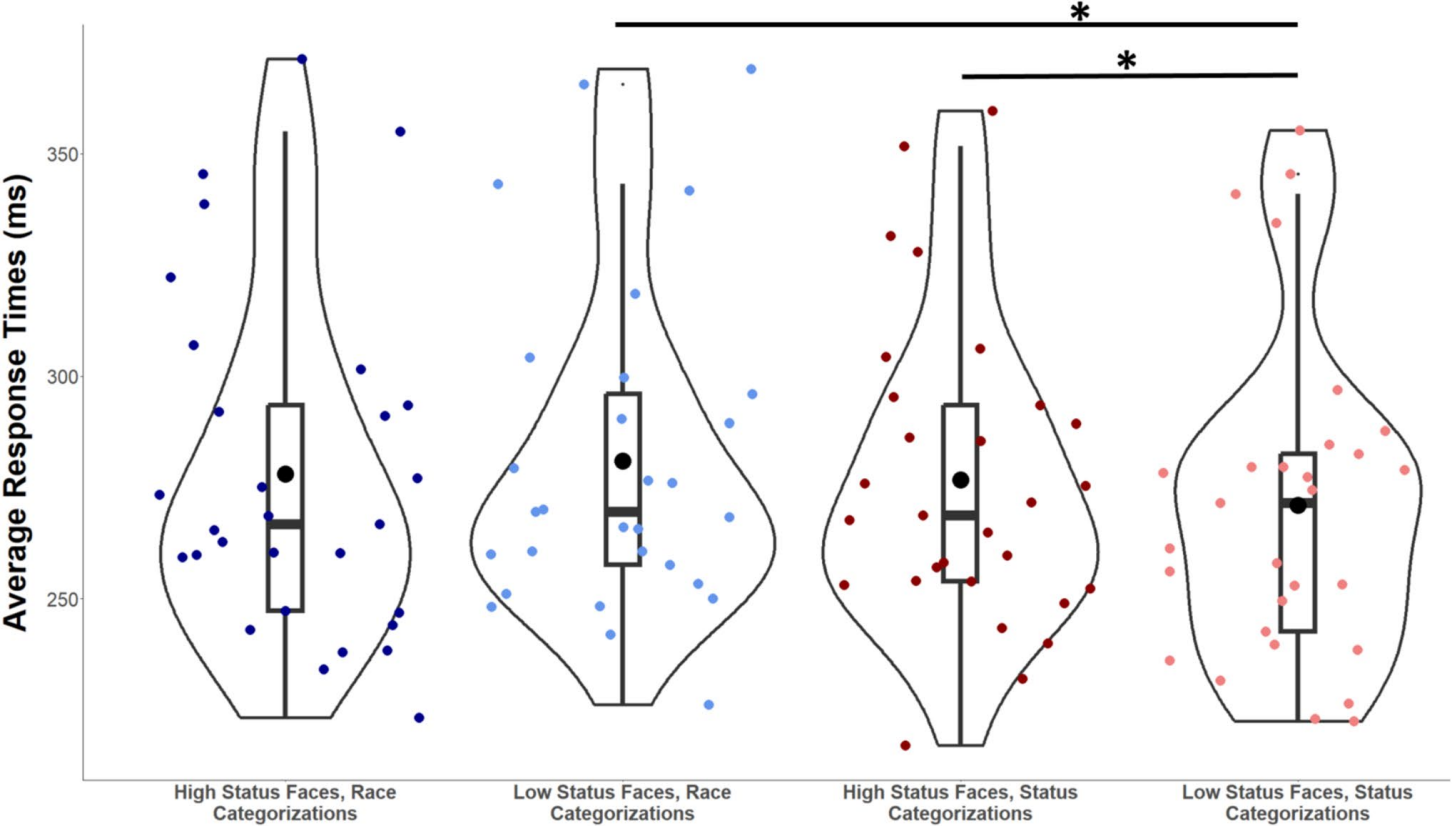
*Categorization speed as a function of ascribed status and categorization task.* Red dots represent the status categorization task, and blue dots represent the race categorization task. The y-axis represents reaction times in ms, and the x-axis represents conditions. Lighter colored dots represent low-status faces, and darker colored dots represent high-status faces. The mean and the 95% confidence interval are displayed as a point estimate and horizontal bar (black dot and line, respectively). The boxes indicate the interquartile range (i.e., the 25th and 75th percentiles of these data). A black line and asterisk denote significant simple differences within the interaction. For graphical depictions of all conditions, see Supplemental Materials 10)

We first examined categorization speed (*M* = 275.222 ms, *SD* = 97.501 ms) as a function of ascribed status, perceived race, and categorization task. There was a significant main effect of categorization task, *b* = 0.021, *SE* = 0.007, *CI*_*95%*_ = [0.007, 0.036], *t*(27.87) = 2.990, *p* = 0.006, such that participants were faster to categorize faces by ascribed status than by perceived race (note that all inferential statistics are reported in Tables S1–S11 in Supplemental Materials 9). There was also a significant interaction between ascribed status and categorization task, *b* = −0.040, *SE* = 0.010, *CI*_*95%*_ = [− 0.054, − 0.014], *t*(26.74) =  − 3.332, *p* = 0.003. This interaction was driven by significantly slower response times to categorize low-status faces by race relative to categorizing low-status faces by status, *b* = 0.039, *SE* = 0.009, *CI*_*95%*_ = [0.021,0.056], *t*(49.22) = 4.355, *p* < 0.001. Participants were also significantly slower to categorize high-status faces compared to low-status faces within the status categorization blocks, *b* = 0.023, *SE* = 0.007, *CI*_*95%*_ = [0.010,0.037], *t*(53.89) = 3.409, *p* = 0.001 (Fig. 2). All other main effects and interactions were nonsignificant, *p* > 0.24.

### ERP amplitudes

**Fig. 3:**
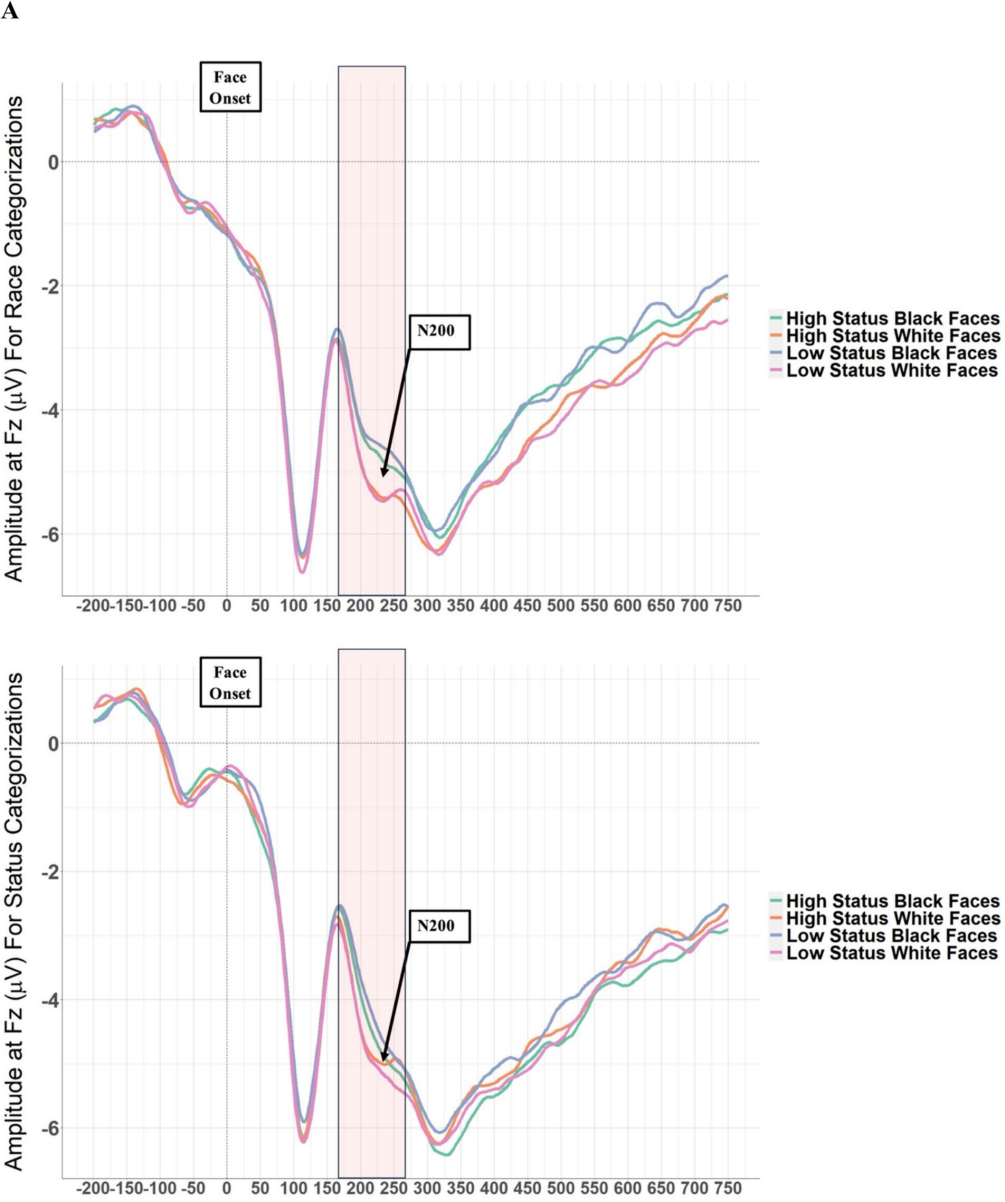

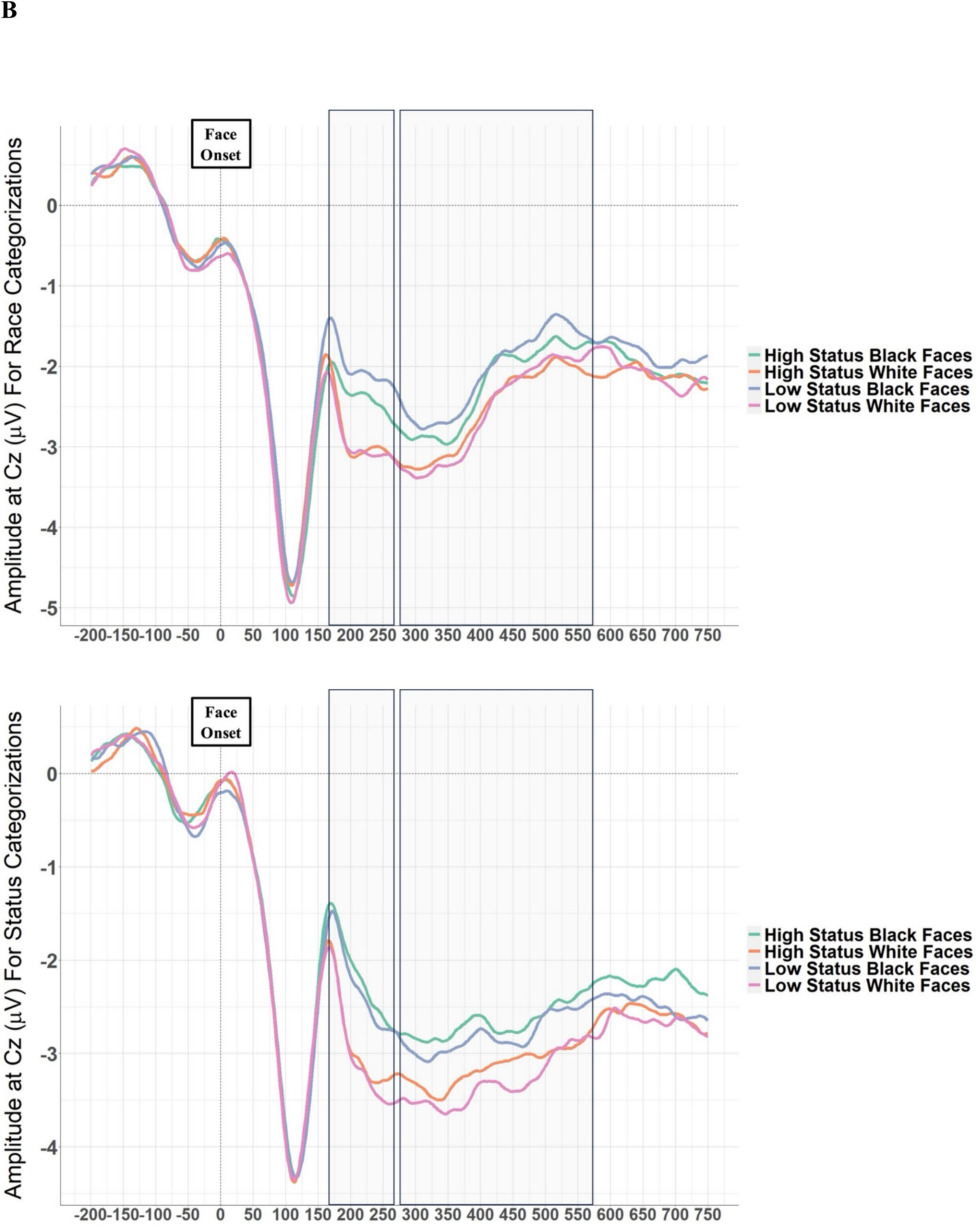

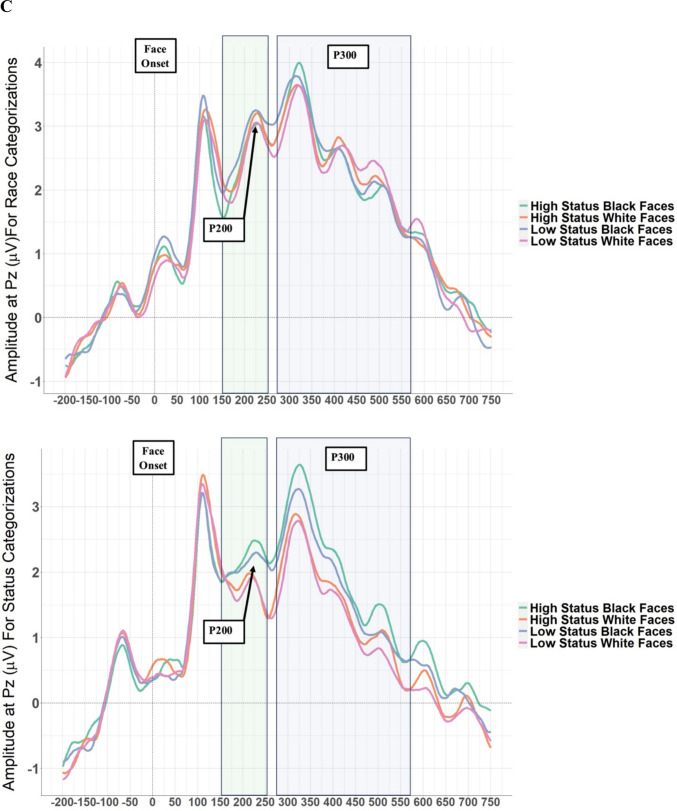
*Grand Average Waveforms.* Grand average waveforms at central electrode sites, from top to bottom Fz (C21; panel **A**), Cz (A1; panel **B**), and Poz (A21; panel **C**) as a function of ascribed status, perceived race, and categorization task. Turquoise lines represent perceived high-status Black faces; lavender lines represent perceived low-status Black faces, high-status White faces are represented by orange lines, and magenta lines represent low-status White faces. The y-axis represents mean amplitudes in µV, and the x-axis reflects time during the measurement windows. The red highlight corresponds to the measurement windows of the N200 component (exploratory), the green highlight corresponds to the P200 measurement window, and the blue highlight represents the measurement window for the P300 component. The grey boxes indicate the time windows for the P200 and P300 at Cz, which was not the maximal location for either ERP component. Average waveforms for race categorizations are displayed first for each corresponding measurement site, followed by the average amplitudes during status categorizations for each corresponding electrode

We next examined ERP amplitudes for the P200 and P300 as a function of ascribed status, perceived race, and categorization task (Fig. 3). Because our analyses of the N200 and N170 components were exploratory, we have included the N200 and N170 results in the supplemental materials (see Supplemental Materials S4 and S5, respectively). Note that all inferential statistics are reported in Tables S1–S11 in Supplemental Materials 9 and include JZS Bayes Factors for ERP analyses.

### P200

**Fig. 4:**
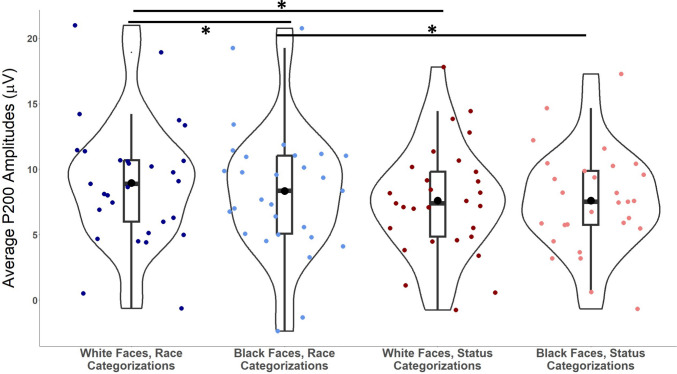
*P200 amplitudes as a function of perceived race and categorization task. *Red dots represent status categorization, and blue dots represent race categorization. The y-axis represents average waveform amplitudes in µV, and the x-axis represents conditions. Lighter-colored dots represent perceived Black faces, and darker-colored dots represent perceived White faces. The mean and the 95% confidence interval are displayed as a point estimate and horizontal bar (black dot and line, respectively). The boxes indicate the interquartile range (i.e., the 25th and 75th percentiles of these data). The black lines between conditions denote significant simple differences within the interaction. For graphical depictions of all conditions, see Supplemental Materials 10)

We first examined P200 amplitudes (*M* = 8.119 µV, *SD* = 4.487 µV) to assess how ascribed status, perceived race, and categorization task impacts selective attention to salient categories. Results revealed a significant main effect of categorization task, *b* = 1.047, *SE* = 0.294, *CI*_*95%*_ = [0.471,1.624], *t*(28) = 3.561, *p* = 0.001, such that P200s were greater when participants categorized perceived race than ascribed status (Fig. 4). Additionally, there was a significant interaction between perceived race and categorization task, *b* = 0.599, *SE* = 0.226, *CI*_*95%*_ = [0.155, 1.043], *t*(28) = 2.645, *p* = 0.010 (Fig. 4). P200 amplitudes were greater for race categorization relative to status categorization for both perceived Black, *b* = 0.748, *SE* = 0.315, *CI*_*95%*_ = [0.130, 1.366], *t*(36.643) = 2.373, *p* = 0.023, and White faces, *b* = 1.347, *SE* = 0.315, *CI*_*95%*_ = [0.729, 1.964], *t*(36.643) = 4.274, *p* < 0.001. Additionally, although P200 amplitudes were greater in general when participants attended to perceived race (categorized by race) than ascribed status (categorized by status), contrary to our pre-registered hypotheses, P200 amplitudes were greater for perceived White relative to perceived Black faces when participants categorized faces by perceived race, *b* = 0.605, *SE* = 0.221, *CI*_*95%*_ = [0.172, 1.039], *t*(49.376) = 2.740, *p* = 0.009. All other main effects and interactions for the P200 were not significant, *p* > 0.053.

### P300

**Fig. 5:**
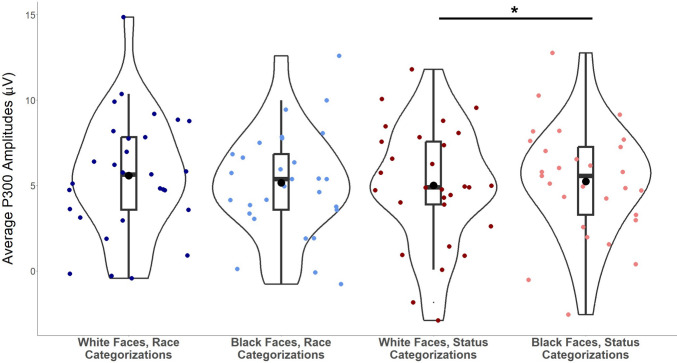
*P300 amplitudes as a function of perceived race and categorization task.* Red dots represent status categorization, and blue dots represent race categorization. The y-axis represents average waveform amplitudes in µV, and the x-axis represents conditions. Lighter-colored dots represent perceived Black faces, and darker-colored dots represent perceived White faces. The mean and the 95% confidence interval are displayed as a point estimate and horizontal bar (black dot and line, respectively). The boxes indicate the interquartile range (i.e., the 25th and 75th percentiles of these data). A black line and asterisk denote significant simple differences within the interaction. For graphical depictions of all conditions, see Supplemental Materials 10)

We next examined P300 amplitudes (*M* = 5.250 µV, *SD* = 3.325 µV) to assess how ascribed status, perceived race, and categorization task impacts evaluative processes. Again, results yielded a significant interaction between perceived race and categorization task, *b* = 0.664, *SE* = 0.190, *CI*_*95%*_ = [0.292, 1.036], *t*(28) = 3.495, *p* = 0.002 (Fig. 5). Unlike the P200, the difference in perceived race occurred not when participants were explicitly asked to categorize/focus on race, but when they were asked to categorize/focus on status. Specifically, there was significantly greater P300 amplitudes to perceived Black faces relative to perceived White faces during status categorizations, *b* = 0.430, *SE* = 0.184, *CI*_*95%*_ = [0.070, 0.790], *t*(46.04) = 2.340, *p* = 0.024. Contrary to our pre-registered P300 hypotheses, we did not observe ascribed status effects, and all other main effects and interactions within the P300 were non-significant, *p* > 0.137.

### Functional connectivity

**Fig. 6:**
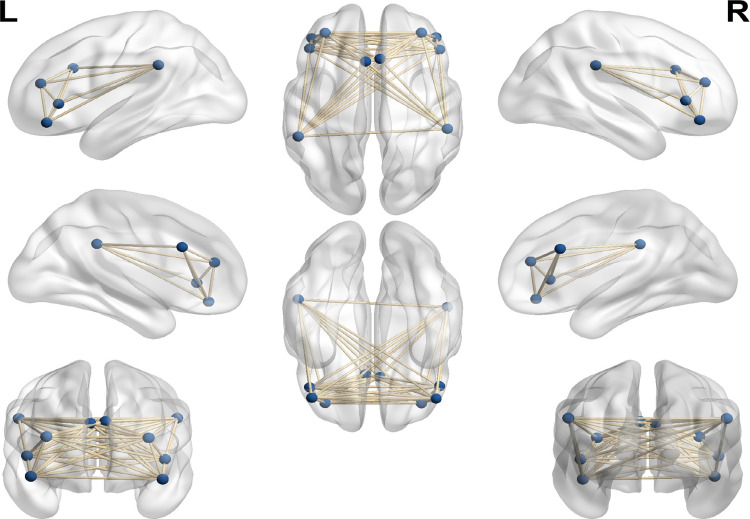
*Predefined attention/executive function network consisting of the IPS, VLPFC, DLPFC, and DACC.* The figure depicts medial, lateral, and frontal views of the left and right hemispheres, as well as ventral and dorsal views of both hemispheres. The blue spheres represent each node within the attention/executive function network, and the beige lines represent the edges, or connections between each node

To further probe the extent to which neural structures underlying attention/executive function and social cognition/evaluation of perceived race, ascribed status, and task work in concert during categorization, we ran a series of pre-registered exploratory network connectivity modularity analyses. Below, we present select modularity functional network analyses for the attention/executive network (Fig. 6) and for the evaluative/social cognitive network (Fig. 8). Because the effects were largely similar across the alpha, beta, and gamma bands for each network, only results for the alpha band are presented in the main manuscript (see supplemental materials for effects in the beta and gamma networks for the attention/executive function network, S6, and for the evaluative/social cognitive network, S7). Across these networks, unlike the ERP results, the ascribed status consistently shaped network connectivity in the attention/executive function network and the social cognition/evaluation network.

### Alpha connectivity in the attention/executive function network

**Fig. 7:**
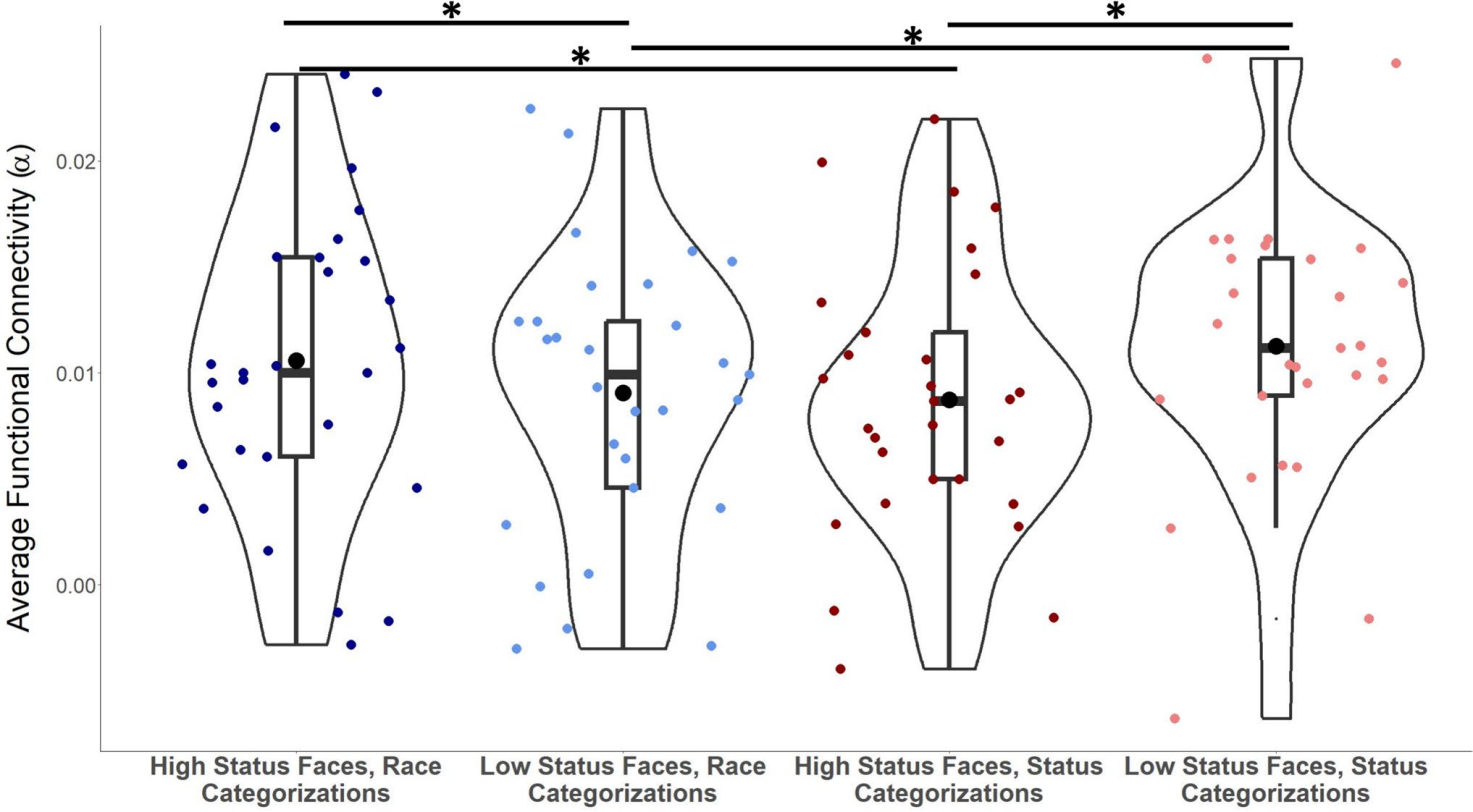
*Functional α connectivity within the attention/executive function network as a function of ascribed status and categorization task.* Red dots represent the status categorization task, and blue dots represent the race categorization task. Lighter colored dots represent low-status faces, and darker colored dots represent high-status faces. The y-axis represents average α functional connectivity, and the x-axis represents conditions. The mean and the 95% confidence interval are displayed as a point estimate and vertical bar (black dot and line, respectively). The boxes indicate the interquartile range (i.e., the 25th and 75th percentiles of these data). A black line and asterisk denote significant simple differences within the interaction. For graphical depictions of all conditions, see Supplemental Materials 10)

Analyses of α band network connectivity (*M* = 0.010, *SD* = 0.0072) indicated a significant cross-over interaction between ascribed status and categorization task,* b* = 0.004, *SE* = 0.001, *CI*_*95%*_ = [0.002, 0.006], *t*(196) = 3.889, *p* < 0.001. Specifically, this interaction revealed greater α connectivity to low- relative to high-status faces when participants were categorizing by ascribed status, *b* =  − 0.003, *SE* = 0.0007, *CI*_*95%*_ = [− 0.004, − 0.001], *t*(196) =  − 3.349, *p* < 0.001. Conversely, there was greater α connectivity to high-status relative to low-status faces when categorizing by perceived race, *b* = 0.002, *SE* = 0.0007, *CI*_*95%*_ = [0.0007, 0.003], *t*(196) = 2.061, *p* = 0.040. Moreover, greater α connectivity was observed for high-status faces during race categorization relative to status categorization,* b* = 0.002, *SE* = 0.001, *CI*_*95%*_ = [0.0004, 0.0033], *t*(196) = 2.504, *p* = 0.013. The opposite pattern emerged for low-status faces, as greater α was observed for low-status faces when categorizing by status relative to race, *b* =  − 0.002, *SE* = 0.001, *CI*_*95%*_ = [− 0.004, − 0.001], *t*(196) =  − 2.996, *p* = 0.003 (Fig. 7). All other main effects and interactions within this waveband were nonsignificant (*p* > 0.127).

### Alpha connectivity for the social cognitive/evaluation network

**Fig. 8:**
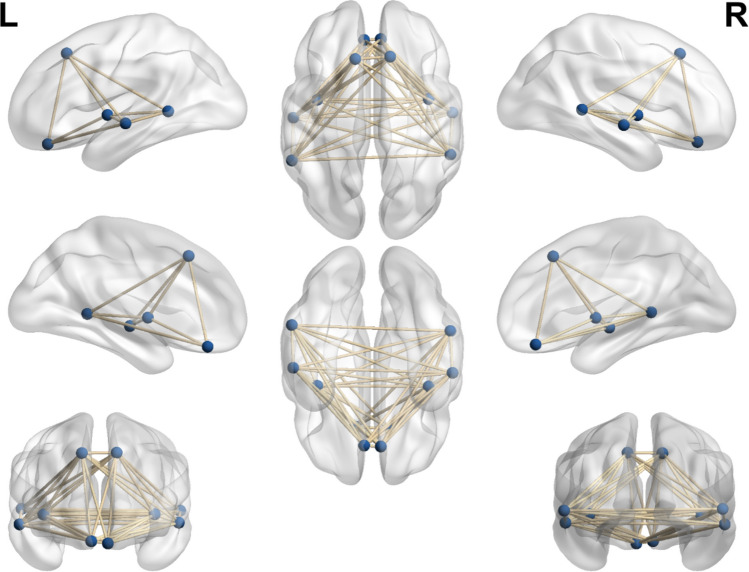
*Predefined social cognitive/evaluative network consisting of the VMPFC, DMPFC, STS, and insula.* The figure depicts medial, lateral, and frontal views of the left and right hemispheres, as well as ventral and dorsal views of both hemispheres. The blue spheres represent each node in the social cognitive/evaluative network, and the beige lines represent the edges, or connections, between nodes

**Fig. 9:**
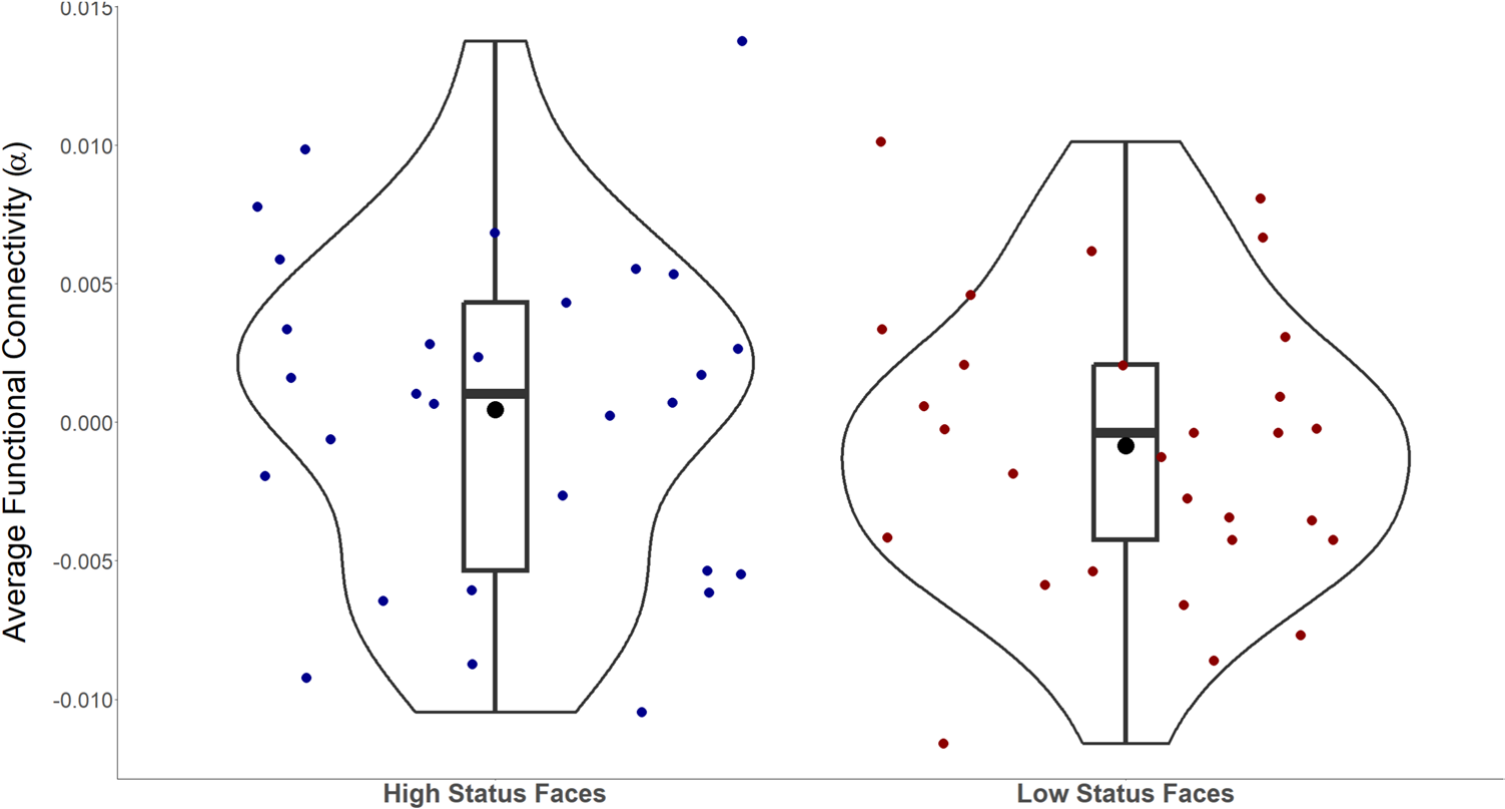
*Functional α connectivity within the social cognition/evaluative network as a function of status.* Red dots represent the low-status faces, and blue dots represent the high-status faces. The y-axis represents average α functional connectivity, and the x-axis represents conditions. The mean and the 95% confidence interval are displayed as a point estimate and vertical bar (black dot and line, respectively). The boxes indicate the interquartile range (i.e., the 25th and 75th percentiles of these data). Results revealed significantly greater coordination within the α frequency band toward high-status faces relative to low-status faces. For graphical depictions of all conditions, see Supplemental Materials 10)

Analysis of α band network connectivity (*M* =  − 0.00019, *SD* = 0.0065) (Fig. 8) revealed a significant main effect of ascribed status in the social cognition/evaluative network (Fig. 9). Greater α connectivity was observed for high- relative to low-status faces, *b* = 0.0013, *SE* = 0.0005, *CI*_*95%*_ = [0.0002, 0.002], *t*(196) = 2.467, *p* = 0.015. All other main effects and interactions were not significant (*p* > 0.204).

## General discussion

Little research has examined how person knowledge (or the interaction between perceptual category cues and person knowledge) influences the temporal unfolding of person perception (Ito & Kubota, 2024), thereby missing a common experience of everyday encounters in which individuals have access to both. The present research found evidence of a possible dissociation (i.e., different results) between ERP responses (i.e., evoked attention and motivation/evaluation) and sustained functional neural network coordination (i.e., attention/executive function and social cognitive/evaluative regions) to perceptual information (perceived race) and person knowledge (ascribed status). Specifically, perceived race evoked in-the-moment differences in P200s and P300s, but ascribed status did not. Instead, ascribed status influenced sustained attention/executive function, and social cognitive/evaluative neural network coordination, whereas perceived race did not. These findings suggest that perceptual information (perceived race) and person knowledge (ascribed status) might influence impression formation in distinct ways: one in an immediate, evoked manner (ERPs, perceptual information) and the other through sustained coordination of functional networks (person knowledge). The differing results are not best understood as indexing a simple temporal dissociation, but rather as reflecting how perceived race (perceptual information) and ascribed status (person-knowledge) differentially influence event-locked neural responses (perceived race) versus distributed network coordination (ascribed status). Additionally, the categorization task modulated both the evoked ERP and neural network coordination, suggesting that task goals direct in-the-moment and sustained responses during person perception.

These findings provide three novel insights. First, we find a dissociation in evoked responses and sustained neural network coordination during person perception. Second, we replicate previous findings of rapid processing of perceived race (Kubota & Ito, 2007, 2014) with the added nuance that these observed differences in selective attention to race change depending on task goals. Finally, extending previous fMRI research, we find that status influences sustained attention and evaluation (Mattan et al., 2018c). We elaborate on each of these points below.

The current ERP findings generally diverge from previous studies regarding race and status perception; therefore, results only partially supported our confirmatory hypotheses. Although we predicted a priori that P200s would differ as a function of perceived race, we expected P200s to be greater for perceived Black faces than for perceived White faces. We did not anticipate that the categorization task would modulate these effects. Instead, we found divergence from previous literature, which typically reports greater P200s to outgroup faces (Ito & Urland, 2003, 2005; Kubota & Ito, 2007, 2017). P200s were larger for perceived White faces than for perceived Black faces when categorizing faces by race. These results may indicate that although categorizing by perceived race relative to status may be especially salient for White U.S. perceivers, leading to greater P200 amplitudes in the race categorization blocks relative to the status categorization blocks, perceived White faces may be especially salient when White U.S. participants are asked to focus on race compared to Black faces when status information is present. In addition, we replicate previous research suggesting that status does not differentially impact evoked early selective attention (Gyurovski et al., 2018), but rather does so in a more sustained manner across the task (Cloutier & Gyurovski, 2014; Cloutier et al., 2012).

In the context of social category processing during non-oddball categorization tasks, the P300 has been associated with stimulus evaluation, with increased amplitudes in response to a stimulus’s motivational or affective salience. Event-related brain potential research has found that perceived race and status impact P300s (Dickter & Bartholow, 2007; Gyurovski et al., 2018; Ito & Urland, 2003, 2005). Therefore, we predicted that perceived race and ascribed status would interact to impact P300 amplitudes, and we did not anticipate that the categorization task would modulate these effects. However, these confirmatory hypotheses were not supported. When asked to categorize perceived race (to focus on race) explicitly, P300s no longer differentiated race as they did with the P200, perhaps suggesting that when asked to focus on race, both perceived Black and White faces were similarly motivationally relevant during later processing. However, when asked to focus on ascribed status (categorize status rather than race), the implicit focal category of perceived race did shape P300s, with greater responses to perceived Black than White faces, providing nuance to previous research that found greater responses to perceived Black faces. Indeed, previous studies have generally found greater P300 amplitudes to members of racial outgroups (Ito & Urland, 2003, 2005; Kubota & Ito, 2007; Willadsen-Jensen & Ito, 2006). However, the current findings reveal greater P300 amplitudes to perceived Black than White faces when participants attended to ascribed status, perhaps because these U.S. participants were less concerned about appearing biased, allowing ingroup/outgroup effects to emerge. In other words, participants may have been hypervigilant during race categorization, yielding both perceived White and Black faces motivationally salient. Future research should seek to replicate and extend these findings by examining how multiple social categories and task goals shape the motivational relevance of perceived race.

Second, contrary to our initial hypotheses, social status did not shape P300 responses. However, no ERP studies have investigated ascribed SES as the specific dimension of manipulated social status (Mattan et al., 2017). Previous neuroimaging and ERP research that identified differential evaluative responses as a function of social status assigned distinct status dimensions to target faces (i.e., moral and financial status; Cloutier & Gyurovski, 2014; Gyurovski et al., 2018). Therefore, in the context of our task, SES did not shape evoked responses. However, ascribed SES was found to shape sustained network coordination during social categorization. Future research should directly compare SES and financial/moral status to investigate how status shapes person perception in the context of other perceived social categories, such as gender.

What does the integration of ERPs and network analyses tell us? While the ERP analyses did not reveal status effects, results from the network analyses may indicate that participants were preferentially attending to and evaluating status as categorization unfolded. Across the entire experiment, ascribed status affected network connectivity in the attention/executive function and social cognition/evaluation networks. Additionally, in contrast to the ERP results, perceived race did not affect these network responses. This suggests that large-scale network coordination is not necessary for processing perceived race during categorization, which may instead be supported by neural responses underlying the P200 and P300.

Ascribed status differentially impacted attention/executive function network coordination depending on the categorization task. When categorizing by ascribed status, participants had greater within-network coordination to low-status faces relative to high-status faces, perhaps because these faces were more similar to their ingroup than high-status faces for this sample of college students with average/low SES (Ratner et al., 2013; Hong et al., 2022; for a discussion of processing same-race individuals with EEG, see Han, 2018). However, when categorizing by perceived race and not explicitly asked to focus on ascribed status, participants had greater within-network coordination for high-status than low-status faces. When focusing on race, high-status individuals appeared to draw greater sustained attention, even when it was irrelevant to the task. Previous research has also found greater attention to high-status targets when people are not explicitly asked to categorize them by status (Cheng et al., 2013; Dalmaso et al., 2011; Jones et al., 2010). These results suggest that perceived status has a sustained influence on attention/executive function network coordination even when status is irrelevant to the task goals, in this case, categorization.

Status effects for the social cognition/evaluation network replicate previous fMRI research on status (Cloutier & Gyurovski, 2014; Cloutier et al., 2012). Previous research found more positive evaluations of high-status individuals compared with low-status individuals (Anderson & Kilduff, 2009; Varnum, 2013). Additionally, when status is conveyed through person knowledge, the VMPFC, a region involved in the generation of affective meaning and positive social evaluation (Cloutier & Gyurovski, 2014; Cloutier et al., 2012; Mattan et al., 2018a) was found to respond to high-status others preferentially (Barth et al., 2020; Cloutier & Gyurovski, 2014). Together, these findings suggest that greater network coordination among social cognitive/evaluative networks in response to high-status faces may index sustained social cognitive engagement associated with positive evaluative response to high-status others (Mattan et al., 2017).

An additional point for the EEG network analyses is essential to consider. Given that the nodes correspond to electrodes and not brain regions, there is a limitation in identifying the contribution of specific neural regions to the effects, for example, the contribution of attentional versus cognitive control processes. The attention network comprised brain regions underlying both attention and executive function. As such, it is challenging to determine whether greater attentional demands drove the effects, if greater cognitive control was activated differently, or if it was a combination of both attention and executive function. Future research could compare findings across changes in attentional versus cognitive control demands (e.g., under cognitive load manipulations or with explicit attentional cueing) or use methods with greater spatial resolution (e.g., fMRI) to tease apart further the contributions of these networks to status processing. Moreover, because EEG network topography can vary across individuals and past work examining EEG network analyses has relied on larger samples (Forbes et al., 2018), future research should seek to replicate the study’s exploratory network findings with a larger sample.

Finally, the obtained findings highlight important future considerations for our understanding of how social status impacts person perception and social categorization. Specifically, previous research has focused on different dimensions of status (e.g., moral status, financial status) and has not considered both implicit and explicit status-based person perception, nor has it assessed the perceivers’ sustained attention. Greater cognitive engagement may occur during explicit consideration of status than during implicit processing. This may depend on the participant’s status or the dimension of status, with greater cognitive engagement when explicitly considering others sharing similar personal characteristics. Indeed, the sample consisted of students with low income (low SES), rendering lower-status individuals potentially more self-relevant. Therefore, the dimensions of status, the relevance of status rank to the participants, and the task need to be further considered in relation to ERP and functional network coordination. Finally, we note that it is essential to consider how different status antecedents, for example knowledge about wealth or competency or other naturalistic cues of status (e.g., clothing, cars, etc.) shape the temporal dynamics of impression formation as well as when social status is identified without the assistance of explicit visual cues (Kumaran et al., 2012; for a longer review of this topic, see Mattan et al., 2017).

## Conclusion

The present study examines how integrating perceptual and knowledge-based social information affects evoked neural responses and functional network coordination during categorization. The present study contributes to a growing body of literature by examining how multiple sources of social information during categorization influence the temporal dynamics of person perception. This is a novel contribution because most EEG research on person perception has often focused on a single social category. Additionally, using multivariate network analysis, these results demonstrate that, although perceived race influences person perception rapidly when encountering someone, it may not have a sustained effect. Status, however, seems to affect how entire networks of the brain differentially coordinate during person perception across time. Given that social information is seldom presented in a vacuum in the real world, our study further underscores the need to consider multiple sources of social information when examining the time course of person perception.

## Supplementary Information

Below is the link to the electronic supplementary material.

Supplementary file1 (DOCX 6118 KB)

## Data Availability

All studies were pre-registered. All data files and analysis scripts for all analyses, including supplemental analyses, are available on the Open Science Framework (https://osf.io/gytj5).
