# Supplementary material for: Dissociable impacts of perceived race and ascribed status in event-related brain potentials and multivariate network activity
Source: Cogn Affect Behav Neurosci. 2026 Feb 5;26(3):1194–221. doi: 10.3758/s13415-026-01401-9 (PMC13260144; doi:10.3758/s13415-026-01401-9)
Supplement: Supplementary file 1 — Supplementary file1 (DOCX 6118 KB) [file 13415_2026_1401_MOESM1_ESM.docx]

# **Supplemental Materials**

# **S1. Power Analyses and Sensitivity Analyses**

# *S1.1 Power Analysis*

We originally conducted a power analysis to account for an individual difference measure. However, due to the COVID-19 pandemic, we were forced to cease data collection. At that time, we collected data from 42 participants (as documented in our pre-registration: <https://osf.io/gytj5>). After applying our pre-registered exclusion criteria, our final sample was reduced to 29 participants. Therefore, we ran an additional power analysis with a sample size of 29 participants and 75 trials per condition (i.e., the minimum number of trials needed to retain a participant in the final sample). We used the default variance parameters in PANGEA (Westfall, 2015; var[error]=.2; var(P*E)=0.04) to estimate power for a 2 (Ascribed Status: high, low) X 2 (Perceived Race: Black, White) x 2 (Categorization Task: Race, Status) within-participants design. Results suggested that 28 participants would be sufficiently powered to detect a significant interaction effect of Status*Race*Task at an effect size as small as *d*=0.15, 1 – *β*=0.80.

## *S1.2 Sensitivity Analysis*

Because of the discrepancy between our reported power analysis and the pre-registration completed after our analyses, we opted to run a post-hoc sensitivity analysis using the SIMR package in R (Green & MacLeod, 2016). To estimate sensitivity, we used a simulation-based approach to determine the minimal effect size for the highest-order interactions observed in our confirmatory analyses. For the P200 and P300, this was conducted on the Race x Task block interaction. The analysis was performed by iteratively adjusting the effect size for each model, testing the significance of either interaction, estimating power using the PowerSim function from SIMR, and finally returning the proportion of times that the effect was significant across 1000 samples. Results from the P200 found that our observed effect size of *b* = 0.59891, was powered at (1 – β=0.764), slightly below 80%. Results from the P300 found that our observed effect size of *b*=0.664, indicating that our results from the P300 were powered at (1 – β=0.925).

## **S2. Exploratory Measures**

*S2.1 Individual Difference Measures*

At the end of the experiment, participants completed a survey block on Qualtrics that included basic demographics, several measures of socioeconomic status (Adler et al., 2000; Mattan et al., 2019), Modern Racism Scale (McConahay, 1986), Evaluative Priming Task (Fazio et al., 1995), Race Contact/Childhood Exposure Scale (Cloutier et al., 2014), and Internal and External Motivation to Respond Without Prejudice (Plant & Devine, 1998). The study was not powered to examine these measures, and they were included as preliminary data for future studies. However, for those interested, all individual difference measures collected are available on OSF (OSF Link: https://osf.io/vkj8r).

*S2.1.1. Subjective and Objective Social Status*

Participants were asked to rate their subjective and objective social status. Subjective socioeconomic status (SES) was assessed using the MacArthur Scale of Subjective Social Status (Adler et al., 2000). The MacArthur scale presents participants with a ladder comprising rungs labeled 1 to 10 in ascending order, where a 1 represents the rank of people who have the lowest standing amongst the general population in the U.S., and a 10 represents the rank of people who have the highest standing among the general population of the U.S.

Following previous recommendations (Oakes & Rossi, 2003) and other work on how perceived social status affects decision-making (Mattan et al., 2020), we used several single-item measures reflecting income, education, and assets. These measures can be used to compute a composite score of objective SES that equally weights income, education, and assets.

*S2.1.2. Modern Racism Scale (MRS)*

Participants were asked to indicate the extent to which they agreed or disagreed with a series of statements on a scale ranging from 1 (*strongly disagree*) to 5 (*strongly agree*). The scale intends to measure White Americans’ racial attitudes towards Black Americans.

*S2.1.3. Evaluative Priming Task (EPT)*

The evaluative priming task (Fazio et al., 1995) is designed to measure evaluative differences using valenced words (Positive, Negative) and faces differing in perceived race (Black, White) as primes. For this, we used an adapted version of the task (Mattan et al., 2019). This version used a different set of faces from the Chicago Face Database (CFD; Ma et al., 2015) such that none of the faces that appeared in the EEG task appeared in the EPT. In each version of the task, participants viewed 10 Black and 10 White face primes, half of which were high-status, and half were low-status. These stimuli were equated on perceived race prototypicality, trustworthiness, threat, dominance, attractiveness, and emotional expression. Color status associations were determined by each face prime’s colored t-shirt, consistent with the task reported in the main text.

In this task, participants were instructed to categorize words as either positive or negative and completed ten practice trials. After the initial 10 trials, participants were informed that they would see a brief image of a face before the word they were to categorize and asked to ignore the image and focus only on categorizing the word as positive or negative. Participants then completed eight additional practice trials before continuing to the main experimental trials without pause. Each trial consisted of a 500ms fixation cross, followed by face prime presentation for 300ms. After the prime presentation, participants had 700ms to categorize the words. Each trial was 1500ms.

*S2.1.4. Interracial Contact*

Lifetime interracial contact was measured by collecting information about the participant’s current and childhood interracial contact (Cloutier et al., 2014, 2017; Kubota et al., 2017; Li et al., 2016). The Interracial Contact Questionnaire (Cloutier et al., 2014, 2017; Kubota et al., 2017; Li et al., 2016) asks participants about the quantity and quality of their contact with members of other racial groups across childhood, adolescence, and adulthood.

*S2.1.5. External and Internal Motivation to Respond without Prejudice (EMS/IMS)*

This questionnaire is designed to assess the extent to which people seek to control the expression of racial prejudice (Plant & Devine, 1998). Participants were instructed to indicate the extent to which they agreed or disagreed with a series of statements describing one’s motivation to be unprejudiced on a 9-point scale ranging from 1 (*strongly disagree*) to 9 (*strongly agree*).

*S2.2. Exploratory ERP Latency Measures*

We also collected ERP component latency as an exploratory measure. Based on previous investigations of social categories using ERPs and categorization tasks (Kubota & Ito, 2007), we did not have strong a priori predictions that component latencies would differ as a function of perceived race, ascribed status, or categorization task.

### *S2.2.1 P200 Latency*

Results yielded significant main effects of perceived race, *b*=-8.031, *SE*=2.549, *CI_95%_*=[-10.585,-0.595], *t*(196)=-2.193, *p*=0.030 and task, *b*=11.688, *SE*=2.549, *CI_95%_=*[3.693,13.683], *t*(196)=3.409, *p*<0.001, indicating that participants elicited P200 peaks more rapidly when categorizing perceived White faces and categorizing faces by perceived status respectively. All other interactions and main effects were not significant, *p*>0.580.

### *S2.2.2 N200 Latency*

Results yielded no significant main effects or interactions at the N200, *p*>0.059.

### *S2.2.3 P300 Latency*

Results yielded no significant main effects or interactions at the P300, *p*>0.085.

*S2.3. Exploratory Accuracy Analyses During Categorization ERP Task*

We assessed accuracy data for the categorization task. We did not have a priori predictions for differences in task accuracy based on target perceived race, ascribed status, or task, as the task was relatively easy, and participants were given ample time to view the faces. We found a significant main effect of categorization task, such that participants were significantly more accurate when categorizing faces by perceived race than by perceived status, *b*=1.184, *SE*=0.139, CI_95%_= [0.886, 1.425], *z*=8.507, *p*<0.001. No other significant main effects or interactions were observed as a function of categorization task accuracy, *p*>0.109.

## *S2.4 Explicit Status Recall*

To examine the role of differences in memory for faces varying in perceived race and social status, we employed an explicit status recall paradigm administered after the categorization task. In this memory task, participants were shown each of the 60 faces that they had seen in the categorization task with their status cues removed. Within each trial, participants viewed a fixation cross for 200ms, followed by the face. Participants were instructed to recall the status of each face as being either high or low in social status using their middle or index fingers on the button box. The faces remained onscreen until participants made their decision. Results are discussed below.

### *S2.4.1 Explicit Status Recall Response Times*

Participants’ log-transformed response times were regressed on perceived race and ascribed social status. Results revealed no significant main effects or interactions, *p*>0.081.

### *S2.4.2. Explicit Status Recall Accuracy Rates*

Participants’ accuracy rates were fitted with a generalized linear model using the GLMER function in R (Bates, Mächler, et al., 2015). Results revealed a significant main effect of ascribed status, *b*=0.242, *SE*=0.098, CI_95%_=[0.049 0.434], *z*=2.457, *p*=0.014, indicating greater explicit status recall for high-status relative to low-status faces. Additionally, there was a significant interaction between ascribed status and perceived race, *b*=0.598, *SE*=0.197, CI_95%_=[0.213,0.984], *z*=3.041, *p*=0.002. Participants were more accurate when categorizing perceived White faces as high status relative to low-status faces, *b*=0.541, *SE*=0.0140, CI_95%_=[ 0.267,0.815], *z*=3.868, *p*<0.001. Additionally, participants were more accurate when categorizing perceived Black faces as low status relative to White faces, *b*=-0.426, *SE*=0.0140, CI_95%_=[-0.699,-0.152], *z*=-3.049, *p*=0.002. These results may indicate memory facilitation for the stereotypical targets (i.e., high status White faces and low status Black faces). All other main effects or interactions were non-significant, *p*>0.198.

## ***S3 Random Effects Determination***

Below, we list the steps to determine random effects for all LMER models. To the extent possible, we allowed for between-participants variance in intercepts and slopes for all within-subject factors (i.e., random effects), as well as the correlations among these random effects. However, sometimes, full models fail to converge or are, in fact, over-fitted (Bates, Kliegl, et al., 2015). If the full model failed to converge or was overfitted, we followed the steps outlined below in the specified order. If the full model converged and was not overfitted (as determined by the PCA procedure described below), we used the full model for our random effects.

In the event of convergence failure and/or an over-fitted full model, we:

1) Increased the max iterations allowed for convergence (up to 100,000)

2) Reset the optimizer used for convergence

3) Removed all correlation parameters from the random effects structure, ran this model, and conducted a PCA on the resulting model to determine how many random effects could be supported by these data, as detailed by Bates and colleagues at <https://arxiv.org/pdf/1506.04967v1.pdf>.

4) Based on the number of redundant dimensions in these data (determined in the previous step), we removed slopes from the random effects structure, starting with random slopes corresponding to higher-order interactions. When choosing between random effects for interactions in the same order (e.g., two two-way interactions), we removed the random slopes that accounted for the least amount of variance in the model from the previous step.

5) Re-ran the PCA on the reduced model for step 4 to ensure that the model was not over-fitted. If the reduced model remained over-fitted (i.e., the model contains components that account for exactly zero variance), we removed random slopes and repeated the PCA until all components accounted for some non-zero amount of variance.

6) Next, we added in all possible correlation parameters, running PCA to avoid overfitting the model.

a) If adding even one correlation parameter reduced the dimensionality of these data, then we did not add any correlation parameters and used the final model from step 5.

b) If it was possible to include some but not all correlation parameters without reducing the dimensionality of these data, we prioritized conserving the largest correlation parameters. The final resulting model from step 6 with added correlation parameters had to result in a significant log-likelihood ratio test when compared to the final reduced model from step 5. Otherwise, we used the simpler model resulting from step 5.

c) If the dimensionality of these data were conserved after including all possible correlation parameters and the model with all possible correlation parameters resulted in a significant log-likelihood ratio test when compared with the final reduced model from step 5, then we pruned the correlation parameters with low values. However, the pruned model had to result in a non-significant log-likelihood ratio test compared to the model with all possible correlation parameters (see the start of step 6). If this was not the case, then we did not prune any correlation parameters from the model.

d) If the dimensionality of these data were conserved after including all possible correlation parameters but the model with all possible correlation parameters resulted in a non-significant log-likelihood ratio test when compared with the final reduced model from step 5, then we used the simpler model resulting from step 5.

***S4 N200***

Following the rapid, selective attention to salient others, differences in how social category members are processed continue at the N200. This negative-going component peaks at frontocentral sites between 200 and 250 ms after viewing a face. For example, perceivers exhibit greater N200 amplitudes in response to faces of their perceived racial ingroup relative to faces of perceived other races (Correll et al., 2006; Dickter & Bartholow, 2007; Ito & Urland, 2003, 2005; Kubota & Ito, 2007; Willadsen-Jensen & Ito, 2006, 2008). Additionally, N200s are greater to one’s own face than to others’ faces (Tanaka et al., 2006) and to famous than unfamiliar faces (Bentin & Deouell, 2000). N200s also vary with the extent to which perceivers individuate a face (Kubota & Ito, 2017), predicting better memory for ingroup than outgroup faces (Lucas et al., 2011). These findings imply that selective attention at the N200 may facilitate perceptual differentiation of faces, which is often stronger for ingroup than outgroup members. This also suggests that N200 responses may index individuation efforts that often preferentially occur in response to ingroup others.

Readers should note that the N200 was pre-registered as an exploratory ERP component. It was included in exploratory analyses because previous person perception research has found that perceived race influences N200s (Kubota & Ito, 2007), yet there has been no N200 research on ascribed status. Because of this, we did not have strong a priori hypotheses about how both perceived race and ascribed status would influence ERPs. The N200 was quantified as the maximal negative amplitude between 170-270ms post-face onset. Results also revealed that the N200 was greater at Cz relative to Poz, *b*=16.247, *SE*=0.520, *CI_95%_*=[15.227,17.267], *t*(665)=31.238, *p*<0.001. Further testing revealed the N200 was maximal over Fz relative to Cz, *b*=2.172, *SE*=0.260, *CI_95%_*=[1.662,2.681], *t*(665)=8.350, *p*<0.001 or Poz, *b*=10.295, *SE*=0.260, *CI_95%_*=[9.785,10.805], *t*(665)=39.588, *p*<0.001 (see Figure 3 in the main manuscript).

### *S4.1 Results*

We examined N200 amplitudes (*M*=-5.709µV, *SD*=2.591µV) in an exploratory fashion to assess how ascribed status, perceived race, and categorization task impacts selective attention that may facilitate later individuation. Results revealed a significant main effect of perceived race, *b*=-0.600, *SE*=0.138, *CI_95%_*=[-0.870,-0.330], *t*(28)=-4.359, *p*<0.001, replicating that N200 amplitudes were greater for perceived White relative to perceived Black faces. All other main effects and interactions were non-significant *p*>0.052.

### *S4.2 Discussion*

The results from the N200 supported our confirmatory predictions and were consistent with previous work that found greater N200s to members of one’s racial ingroup (Ito & Bartholow, 2009; Kubota & Ito, 2007, 2017). These White U.S. participants showed greater N200 amplitudes to perceived White relative to perceived Black faces. Given the absence of current evidence that higher status leads to preferential individuation and the posited role of the N200 in individuation (Kubota & Ito, 2017), it is not surprising that social status did not impact N200s. Furthermore, the lack of significant N200 differences as a function of categorization task is consistent with the existing ERP literature on individuation. Specifically, greater individuation of ingroup members happens rapidly and occurs despite task demands (Kubota & Ito, 2017). Because this pattern of activity occurred regardless of the categorization task or the ascribed status of the face, the results perhaps suggest that, as person perception unfolds, participants process perceived ingroup White faces more deeply, regardless of the category they focus on or the status of the individual.

# **S5 N170**

### *S5.1 Results*

Additionally, we examined how ascribed status, perceived race, and categorization task impacted N170s. Readers should note that these analyses were not pre-registered and are thus exploratory. To do so, we examined how the N170 component, an ERP component associated with face processing, differed as a function of perceived race, ascribed status, and categorization task. The N170 was measured as the peak negative amplitude between 120-220ms post-face presentation. Since the N170 is maximal over parieto-occipital locations, we tested for maximal amplitudes between electrodes PO7 and PO8 (i.e., Biosemi electrodes A10 and B7, respectively). Results revealed that N170 amplitudes were greater at PO7, *b*=-0.715, *SE*=0.174, *CI_95%_*=[-1.056,-0.375], *t*(434)=-4.118, *p*<0.001. As such, the reported analyses were conducted at electrode PO7 (see Figure S1 for grand averages).

Analysis of the N170 revealed significantly greater N170 amplitudes to perceived Black relative to perceived White faces, *b*=0.331, *SE*=0.144, *CI_95%_*=[0.049,0.613], *t*(196)=2.303, *p*=0.022. Additionally, enhanced N170 amplitudes were observed for the ascribed status categorization task relative to when participants were categorizing by perceived race, *b*=0.377, *SE*=0.144, *CI_95%_*=[0.095,0.658], *t*(196)=2.622, *p*=0.009. All other main effects and interactions were not significant, *p*>0.248.

### *S5.2 Discussion*

Our exploratory analysis of the N170 revealed two primary findings. We observed significantly greater N170 amplitudes to perceived Black than perceived White faces. Additionally, we observed enhanced N170 amplitudes when participants categorized faces by ascribed status relative to when they categorized them by perceived race. However, readers should proceed cautiously when interpreting the between-task effects as the nature of the categorization differed. As these analyses were not pre-registered, nor was our study designed to examine the N170 component, we cautiously interpret the N170 findings.

First, N170 amplitudes differed as a function of perceived race, with greater N170 amplitudes observed when participants viewed perceived Black faces relative to perceived White faces. ERP investigations of race perception have been inconsistent regarding differences in N170 amplitudes (Ito & Bartholow, 2009). Some studies have found larger N170 amplitudes when participants process racial ingroup faces, arguing that, because the N170 may track perceptual expertise in face and object processing, processing faces of racial ingroup members may be associated with greater N170 amplitudes (e.g., Ito & Urland, 2003). Other researchers have argued that because N170s are enhanced when configural processing is disrupted, processing faces that are unfamiliar or processing faces that an individual may have less expertise with (e.g., members of racial outgroups (Michel et al., 2006)) may lead to enhanced N170 amplitudes to members of perceived racial outgroups (Ito & Bartholow, 2009; Stahl et al., 2008; Walker et al., 2008). Additionally, some research has found no differences in N170s as a function of perceived race (e.g., Bentin & Deouell, 2000; Caldara et al., 2004; Ofan et al., 2011). Researchers have argued that the inconsistencies in N170 findings are due to task demands or individual differences (see Anzures & Mildort, 2021; Kubota & Ito, 2009; Senholzi & Ito, 2013). For example, Senholzi and Ito (2013) found that when participants were asked to focus only on perceived race, N170s were larger to perceived White faces relative to perceived Black faces. However, N170s were larger to Black faces than to White faces among participants who attended to the unique identity of the faces, suggesting that attention to identity can result in preferential allocation of cognitive resources to outgroup members. In the current study, the addition of ascribed status as unique person knowledge may have increased attention to identity and could have been the reason the current study found greater N170s to perceived Black than perceived White faces. Additional research should explore how task demands and individual differences impact the N170 as a function of perceived race and ascribed social status.

Second, we did not observe differences in N170s as a function of ascribed social status. This finding is at odds with some work that has found larger N170s to high-status individuals relative to low-status individuals (Chiao et al., 2008; Santamaría-García et al., 2015). However, these previous studies have manipulated status perceptually and not based on person-knowledge (e.g., stars; Santamaría-García et al., 2015). Person-knowledge may take longer to process than perceptual information, which may explain the observed null effects. Future research should compare perceptual- versus knowledge-based status manipulations in N170 research.

Third, these N170 results suggest that categorization tasks may differentially impact face processing. Although the current focus of the manuscript is on the within-task comparisons, we do note that N170 amplitudes were enhanced when participants were categorizing based on status relative to when they were categorizing based on perceived race. However, because the nature of the categorization tasks differed in that in one, individuals were focusing on perceptual information. In the other, individuals had to retrieve person-knowledge, we urge caution at over-interpreting this difference uniquely based on the social dimensions being categorized.

|  |
| --- |
| 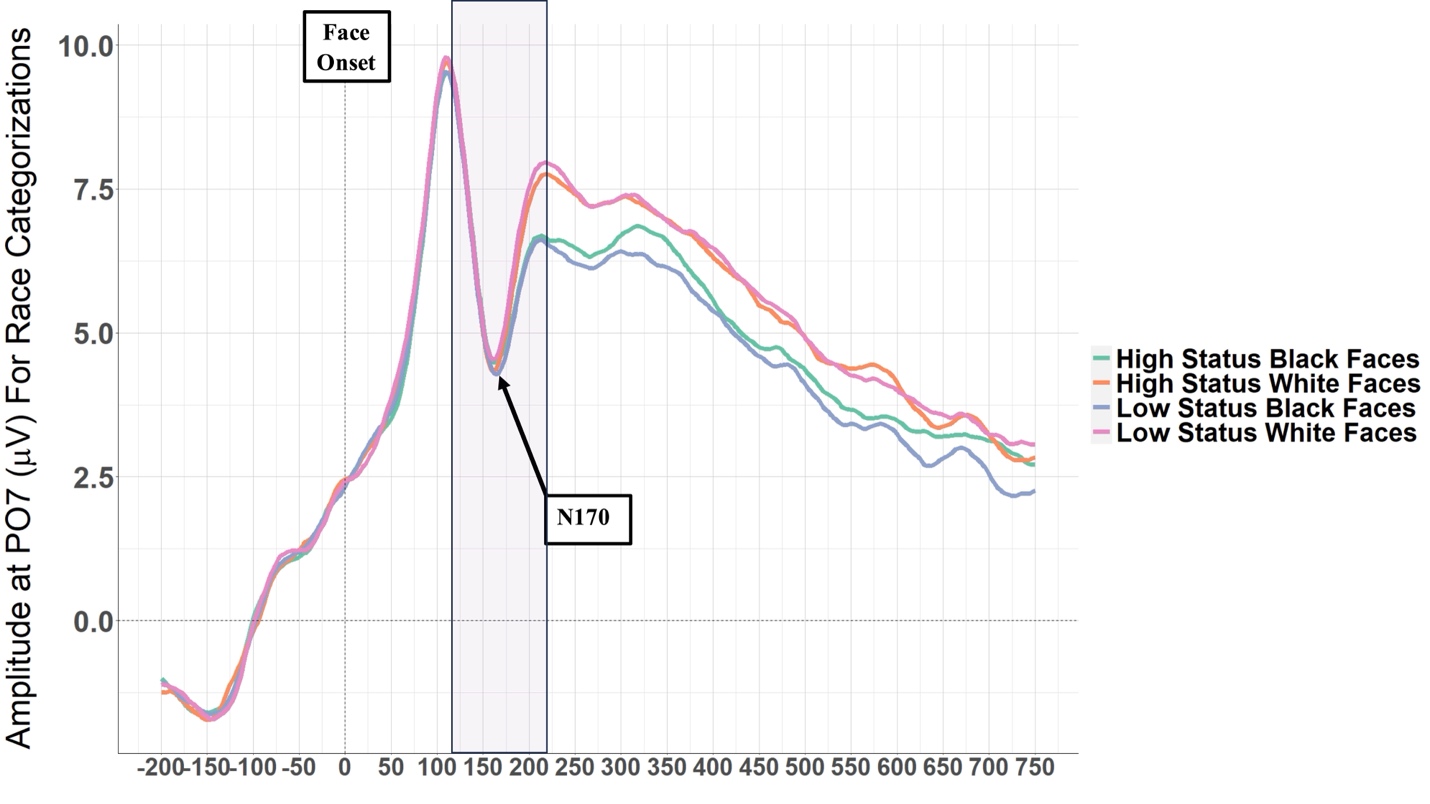  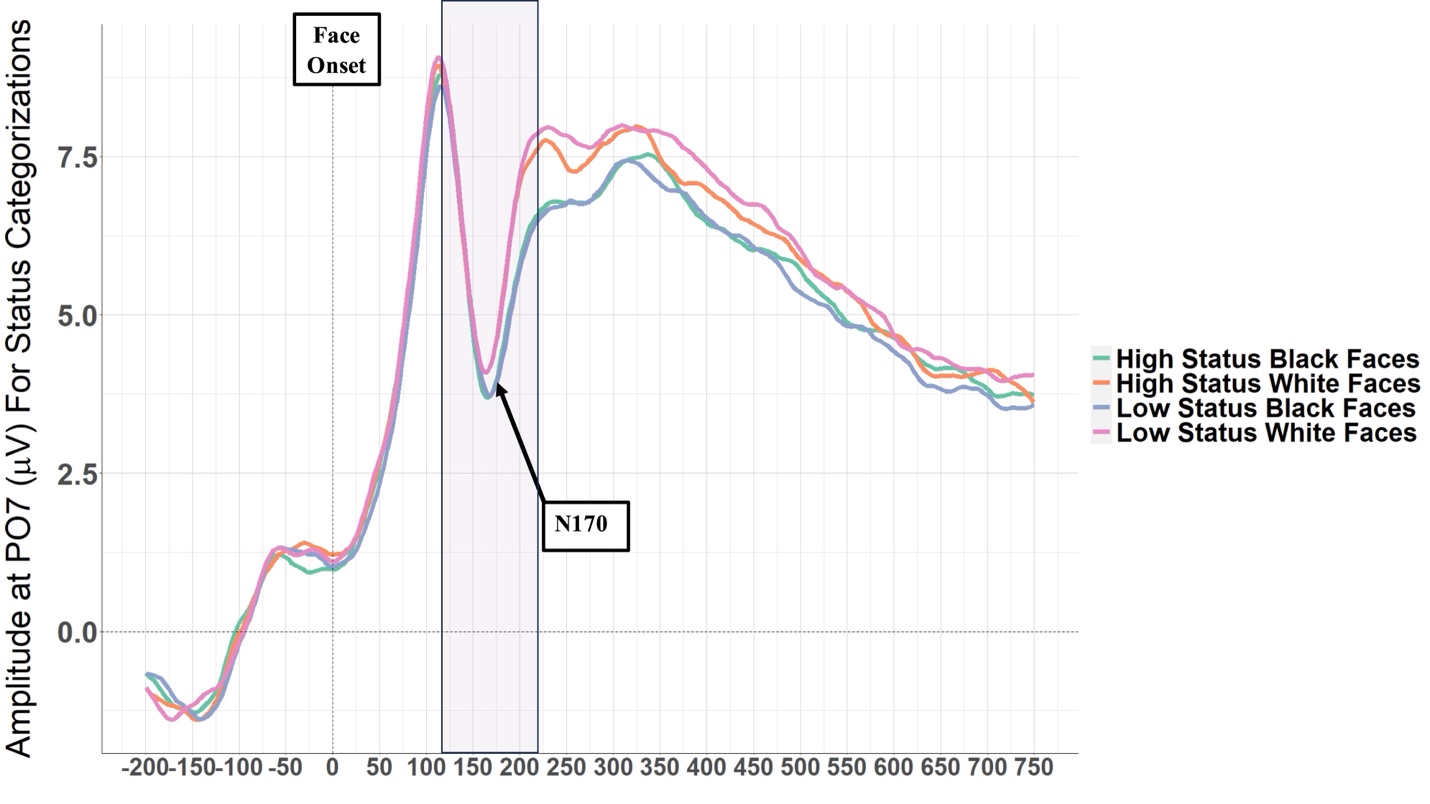 |

#### Figure S1. Grand average waveforms for electrode PO7 as a function of status, perceived race, and categorization task. Turquoise lines represent high-status Black faces; lavender lines represent low-status Black faces, high-status White faces are represented by orange lines, and magenta lines represent low-status White faces. The y-axis represents mean amplitudes in µV, and the x-axis reflects time during the measurement windows. The purple highlight corresponds to the N170 component’s measurement window. Average waveforms for perceived race categorizations are displayed first for each corresponding measurement site, followed by the average amplitudes during status categorizations.

## **S6 Functional Connectivity for the Attention/Executive Function Network for Beta and Gamma**

##### *S6.1 Beta Connectivity for the Attention/Executive Function Network*

Analysis of β band network connectivity (*M*=0.0110, *SD*=0.0075) revealed a significant main effect of perceived race, with greater within-network coordination observed toward perceived White relative to perceived Black faces, *b*=0.0012, *SE*=0.0005, *CI_95%_*=[0.0002,0.002], *t*(196)=2.320, *p*=0.021. Additionally, there was again a significant ascribed status x categorization task interaction, *b*=0.003, *SE*=0.0011, *CI_95%_*=[0.0004,0.005], *t*(196)=2.356, *p*=0.020. This interaction was driven by significantly greater β coordination toward low-status faces relative to high-status faces during status categorization, *b*=0.002, *SE*=0.001, *CI_95%_*=[-0.003,-0.0003], *t*(196)=-2.280, *p*=0.024 (see Figure S2). All other main effects and interactions were not significant (*p*>0.117).

| 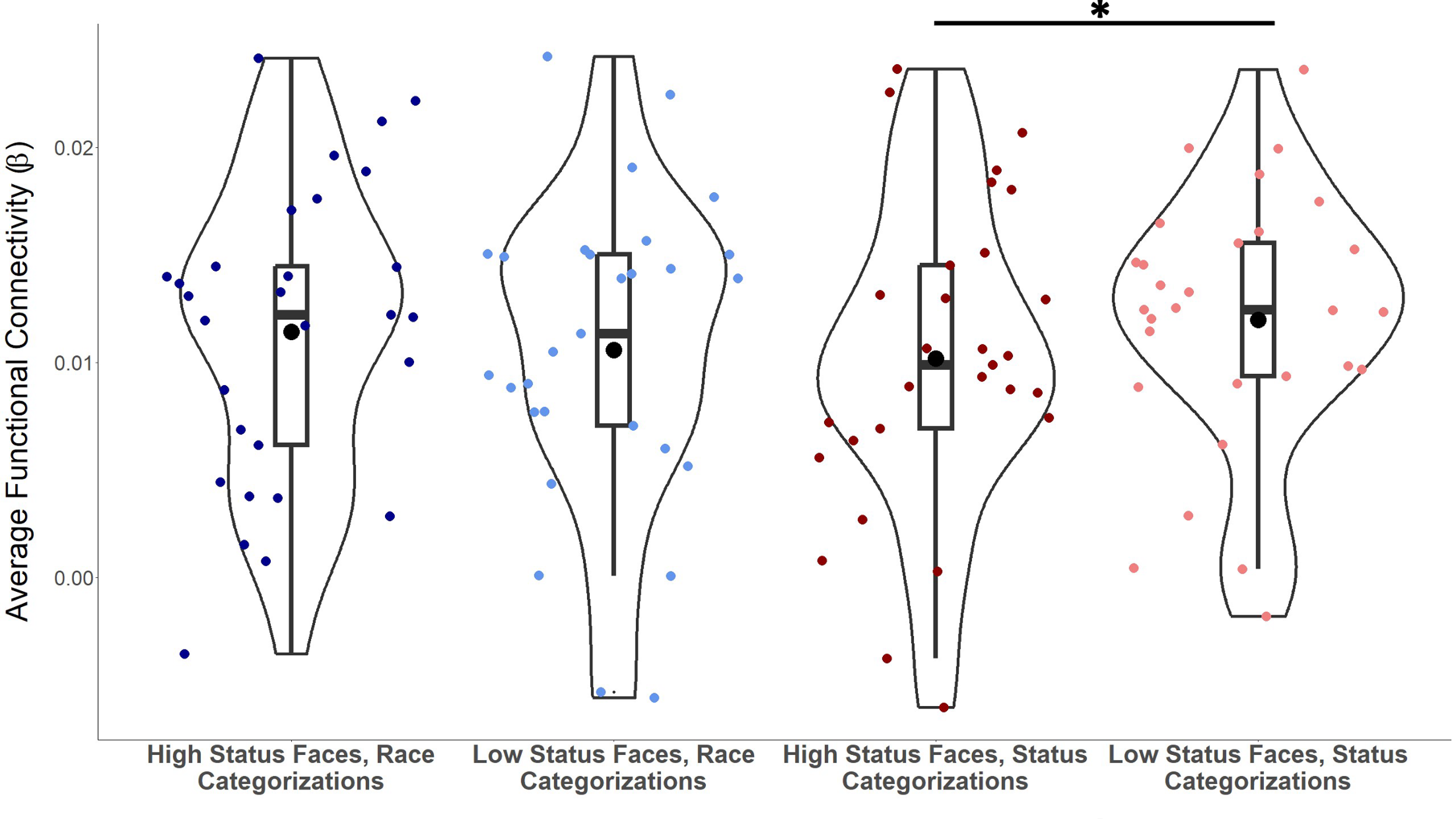 |
| --- |

*Figure S2. Functional β connectivity within the Attention/Executive Function Network as a Function of Ascribed Status and Categorization Task.* Red dots represent the status categorization task, and blue dots represent the race categorization task. Lighter colored dots represent low-status faces, and darker colored dots represent high-status faces. The y-axis represents average *β* functional connectivity, and the x-axis represents conditions. The mean and the 95% confidence interval are displayed as a point estimate and vertical bar (black dot and line, respectively). The boxes indicate the interquartile range (i.e., the 25^th^ and 75^th^ percentiles of these data). A black line and an asterisk denote significant simple differences within the interaction.

##### *S6.2 Gamma Connectivity for the Attention/Executive Function Network*

Analysis of γ band network connectivity (*M*= 0.0114, *SD*=0.0085) revealed a significant ascribed status x categorization task interaction within the γ band, *b*=0.003, *SE*=0.001, *CI_95%_*=[0.0003,0.005], *t*(196)=2.192, *p*=0.030. Similar to the task x status interaction in the α band, the ascribed status x categorization task interaction in the γ band was driven by significantly greater γ coordination toward low-status faces during status categorizations, *b*=-0.002, *SE*=0.001, *CI_95%_*=[-0.004,-0.001], *t*(196)=-2.736, *p*=0.007 (see Figure S3). All other main effects and interactions were not significant (*p*>0.051).

| 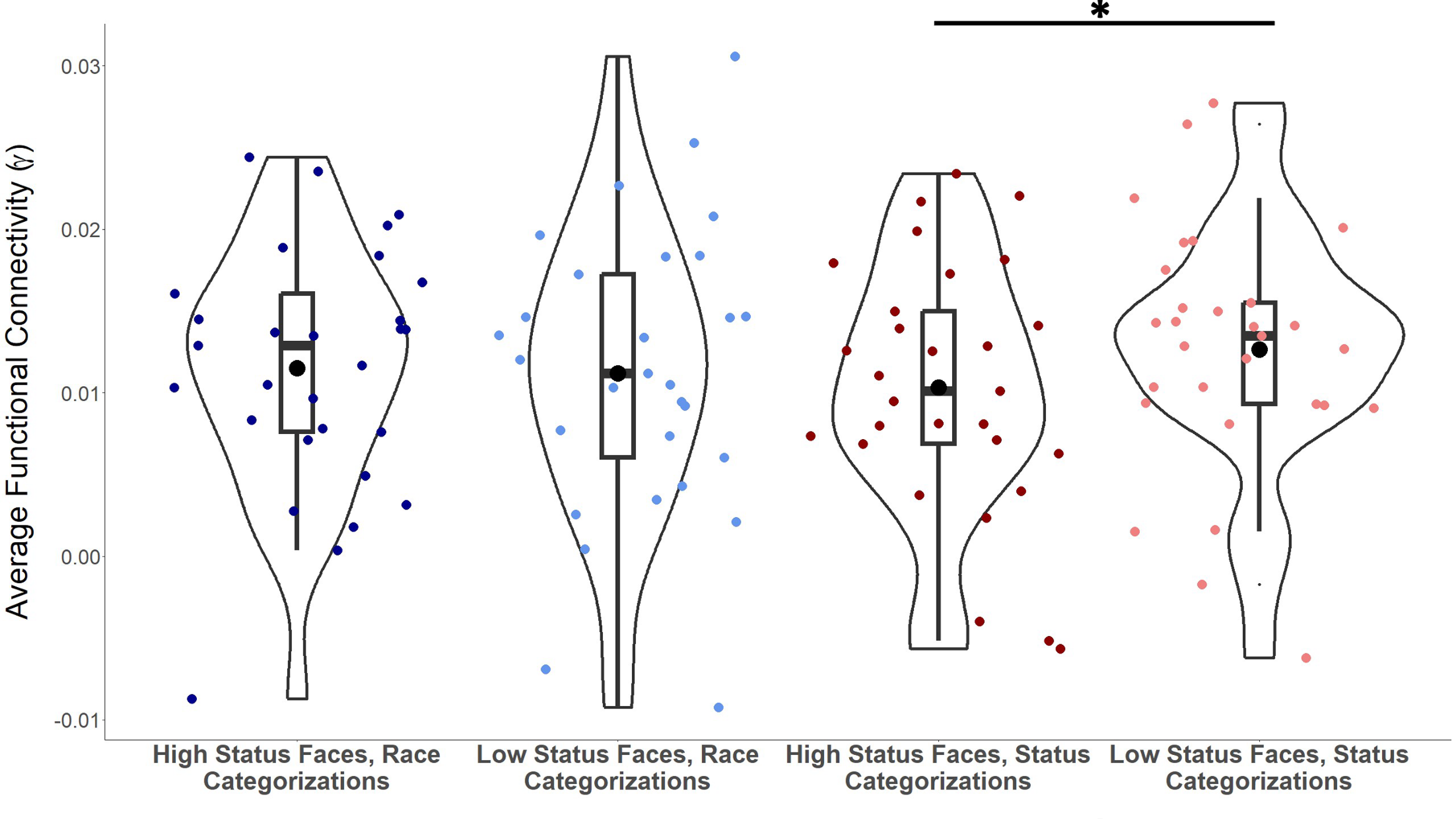 |
| --- |

*Figure S3. Functional* γ *connectivity within the Attention/Executive Function Network as a Function of Status and Task.* Red dots represent the status categorization task, and blue dots represent the race categorization task. Lighter colored dots represent low-status faces, and darker colored dots represent high-status faces. The y-axis represents average γ functional connectivity, and the x-axis represents conditions. The mean and the 95% confidence interval are displayed as a point estimate and vertical bar (black dot and line, respectively). The boxes indicate the interquartile range (i.e., the 25^th^ and 75^th^ percentiles of these data). A black line and an asterisk denote significant simple differences within the interaction.

## **S7 Functional Connectivity for the Social Cognition/Evaluation Network for Beta and Gamma**

##### *S7.1 Beta Connectivity for the Social Cognition/Evaluation Network*

Analysis of β band network connectivity (*M*=-0.00065, *SD*=0.0065) revealed a significant main effect of ascribed status in the β band across the evaluative network, such that greater β connectivity was observed for high- relative to low-status faces, *b*=0.0013, *SE*=0.0005, *CI_95%_*=[0.0002,0.0022], *t*(196)=1.992, *p*=0.048. All other main effects and interactions were not significant (*p*>0.204).

| 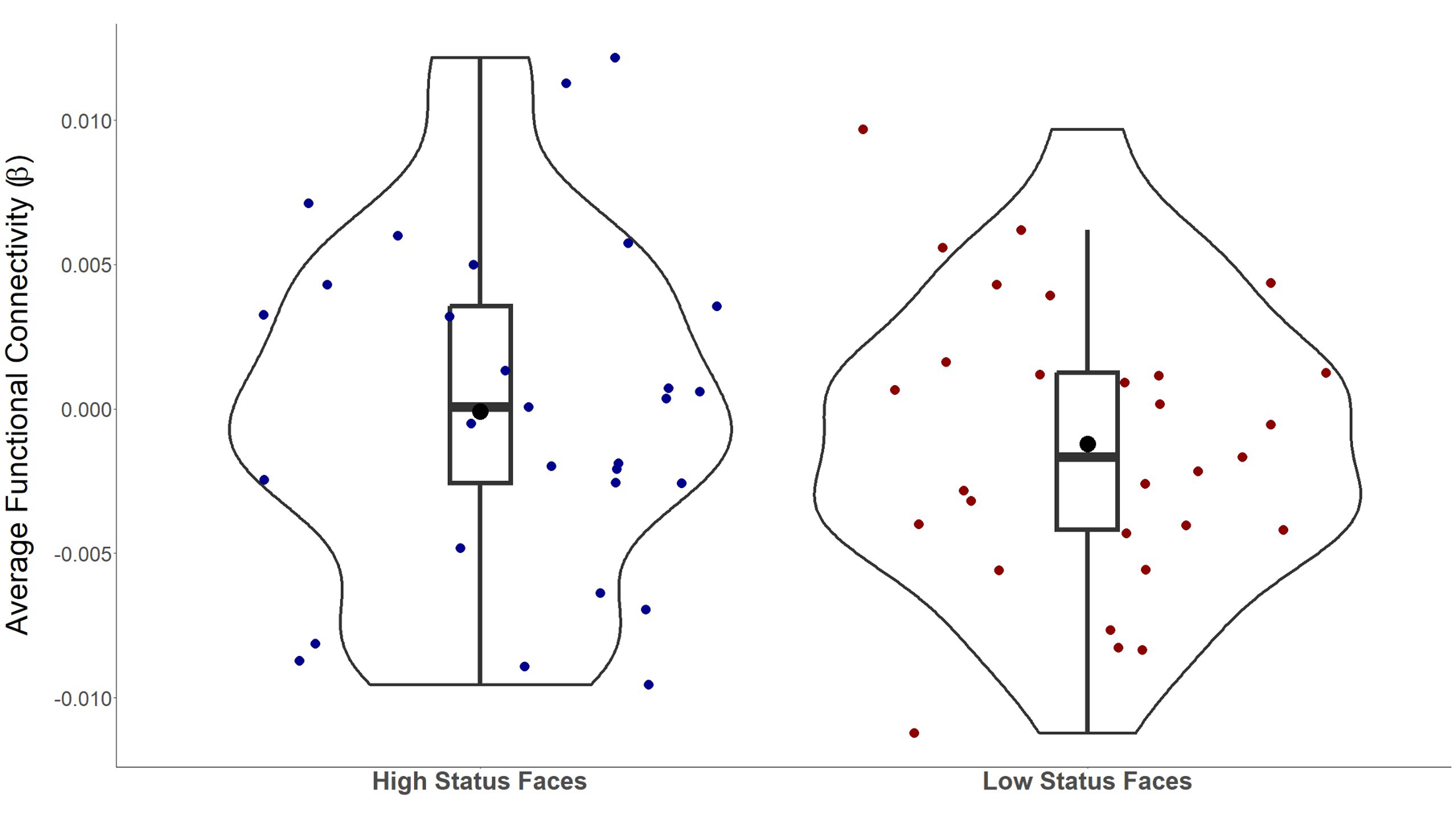 |
| --- |

*Figure S4. Functional* β *connectivity within the Social Cognition/ Evaluative Network as a Function of Status and Task.* Red dots represent the low-status faces, and blue dots represent the high-status faces. The y-axis represents average β functional connectivity, and the x-axis represents conditions. The mean and the 95% confidence interval are displayed as a point estimate and vertical bar (black dot and line, respectively). The boxes indicate the interquartile range (i.e., the 25^th^ and 75^th^ percentiles of these data). Results revealed significantly greater coordination in the β frequency band toward high-status faces than toward low-status faces.

##### *S7.2 Gamma Connectivity for the Social Cognition/Evaluation Network*

Analysis of γ band network connectivity (*M*=-0.00084, *SD*=0.0063) failed to yield any significant main effects or interactions (*p*>0.114).

## **S8 Pre-registration Amendments**

| **Pre-registration Name** | **Date** | **Stage (e.g., before data collection)** | **Purpose/Changes** | **OSF Link** |
| --- | --- | --- | --- | --- |
| **Original Submission** | **10/1/2018** | **Before Data Collection** | **First Pre-registration of study** | [**https://osf.io/7qagr/register/565fb3678c5e4a66b5582f67#q21**](https://osf.io/7qagr/register/565fb3678c5e4a66b5582f67#q21) |
| **2^nd^ Amendment** | **10/16/2018** | **Before Data Collection** | **“We have created this second registration for this project to correct two errors we later found in the original registration (https://osf.io/7qagr/register/565fb3678c5e4a66b5582f67#q21). The first change is that we are updating our measured variables section to include another citation to more accurately represent the face stimuli that we are using in this study. The second change was to correct a small grammatical error in the last paragraph of the Study Design section. We are making these changes prior to the onset of data collection.”** | **https://osf.io/ga4cx** |
| **3^rd^ Amendment** | **05/19/2019** | **Before Data Collection** | **“This third registration serves as an amendment to the second version of the registration found here (https://osf.io/ga4cx). We are making this change to accommodate several design changes in the timing and training for the main categorization task. Specifically, we have updated the way in which we are defining socioeconomic status in the study, as well as updating the questionnaire which we are using to assess current and childhood interracial contact. We are doing this prior to the collection of any data.** | **https://osf.io/wxn68** |
| **4^th^ Amendment** | **10/29/2019** | **Before Data Collection** | **“Note**** This fourth amendment makes a slight change in the description of the categorization task. Previously, we wrote that practice trials would carry over immediately to the main categorization blocks without pause. Further, we have decided to exclude attention checks from the study. These changes were implemented prior to data collection. “** | **https://osf.io/ufhyz** |
| **5^th^ Amendment** | **5/22/2020** | **Before Data Collection Was Complete** | **“The primary difference in this registration is our dropping of the IMS/EMS variable from our confirmatory analysis. This decision was made prior to cleaning or analyzing the existing EEG data. Additionally, we include details for how we will structure random effects for our analyses.”**  **More Detailed Description of Changes:**  **Amendment 5: In light of the global COVID-19 pandemic, we have decided to cease data**  **collection. We will now focus our confirmatory analysis on how the intersection of race**  **and status influence ERP components and drop the EMS/IMS variables from our confirmatory analysis.**  **This decision was made prior to cleaning or analyzing the existing EEG data. We initially powered our project to explore how the individual difference in**  **EMS influences selective attention to race and status. When we drop this individual difference, we are highly powered to explore how the intersection of race and status influence ERP components. We conducted another power analysis that used the default variance parameters in PANGEA (var[error] = .667) to estimate power for a 2 (Status:**  **high, low) x 2 (Race: Black, White) x 2 (Categorization Task: Race, Status) within-**  **participants design. These results suggest that a sample of 38 participants is sufficiently**  **powered to detect a significant interaction effect of Task*Status*Race at an effect size as**  **small as d=0.15, 1 – β = 1. Additionally, we have included details in our analysis plan**  **regarding how we will structure random effects in our analyses.**  **Amendment 5: Due to the COVID19 pandemic, we were forced to cease data collection.**  **At the time of submission, we collected data from 42 participants before exclusions**  **The study will continue until we reach a sample size of 135 usable participants (see**  **‘Sample Size Rationale’ section).** | **https://osf.io/qaeb3** |
| **6^th^ Amendment** | **2/3/2021** | **Data Collected** | **“This amendment serves to clarify some of our data cleaning procedures in our pipeline. Additionally, we propose a set of exploratory analyses using a less conservative threshold for usable trials (see description section).”**  **More Detailed Description of Changes:**  **“Amendment 6: In this amendment we are proposing the following changes to this pre-**  **registration:**  **1. Amending the data cleaning criteria to reflect the exact criteria that we employed during our data cleaning pipeline.**  **2. Proposing a set of exploratory ERP analyses using a less conservative threshold of usable**  **trials such that participants who have at least 50 usable trials for each condition would be**  **included in the interest of statistical power. Based on previous literature, 50 trials will allow us to detect 0.75 μV.**  **Amendment 6: After following our previously defined cleaning and exclusion.**  **Criteria in Amendment 5, we were left with a final sample of 26 usable participants for our**  **confirmatory analysis. We will also run a set of exploratory analyses using a less**  **conservative threshold of usable trials per condition resulting in an exploratory**  **sample size of 30 participants.** | [**https://osf.io/xjyev**](https://osf.io/xjyev)  **Notes: We implemented these cleaning procedures to adhere to updated best practices in ERP data pre-processing. Data analysis of the existing data were reanalyzed using these procedures.** |
| **7^th^ Amendment** | **10/7/2021** | **Data Collected**  **Confirmatory ERP analyses Run**  **Exploratory Network Analyses Run** | **Amendment 7: In this amendment, we are proposing the following changes to this pre-registration:**  **1. Proposing a set of exploratory analyses that examine connectivity between networks that could potentially underly impression formation of the integration of perceptual and knowledge-based person information.**  **As of Amendment 7, we have analyzed both the confirmatory and exploratory samples (see Amendment 6) of data. We are proposing a set of**  **network analyses as exploratory, yet possibly complementary analyses that we wish to run.** | **https://osf.io/ye2u6** |
| **8^th^ Amendment** | **6/3/2024** | **Data Collected**  **Confirmatory ERP analyses Run**  **Exploratory Network Analyses Run** | **Amendment 8: In this amendment we are proposing the following changes to this pre-**  **registration:**  **1. Updating our power analysis section to reflect our final sample size and minimal trial**  **amount so it is consistent with our final sample size.**  **2. Supplementing our updated power analysis with a sensitivity analysis using the highest**  **order results observed from the P200 and P300.**  **3. Referencing section 23 on page 9 of this pre-registration when discussing networks of**  **interest for the network analysis.**  **Amendment 8: Implementation of the trial-by-trial interpolation procedure allowed us to**  **retain an additional two subjects, leaving us with a final sample of 28 participants.** | **https://osf.io/kf64h**  **Largely a power section update:**  **Note: We realized our analyzed sample was less than we pre-registered needing in Amendment 5 (n=35) based on our previous power analysis. However, we realized that our sample of 28 participants was sufficiently powered, it was just that 35 participants was powered well above the 80% power required.**  **Additionally, we implemented a new trial-by-trial interpolation cleaning procedure to adhere to updated best practices in ERP data pre-processing. Data analysis of the existing data were reanalyzed using these procedures. Giving us two more participants.** |
| **9^th^ Amendment** | **10/20/24** | **Data Collected**  **Confirmatory ERP analyses Run**  **Exploratory Network Analyses Run**  **Both following Amendment 8** | **1. Edited our EEG cleaning procedures so that EEG data was referenced to the scalp average upon import to EEGLab instead of importing with a mastoid reference and then subsequently re-referenced to the scalp (see section 19).**  **2. Updated our post-hoc sensitivity analyses using the results from the updated cleaning pipeline (see section 9).** | [**https://osf.io/ezm9h**](https://osf.io/ezm9h)  **A reference update:**  **Previous researchers have used a scalp average baseline. To more clearly align our results with previous work on ERPs and person perception of perceptual and knowledge-based cues, we decided to reanalyze the data with a mastoid reference.** |

## **S9 Tables of Reported Inferential Statistics**

Below are tables of the inferential statistics for the localizer (Table S1-S2), reaction time (Table S3-S4), ERP analyses (Table S5-S8), and network analyses (Table S9-S11). Note that significant results are indicated with an asterisk. For the ERP analyses, JZS Bayes Factors are included for all nonsignificant results to compare findings to previous ERP research on perceived race and ascribed status. The JZS Bayes factor provides a quantitative comparison of support for the null hypothesis (H₀) versus the alternative hypothesis (H₁), enabling direct evaluation of evidence for the null (Rouder & Morey, 2012; Rouder et al., 2009). Values above 1 reflect greater support for H₀, whereas values below 1 favor H₁. JZS Bayes factors are reported for all nonsignificant results, assuming an r scale parameter of 1. Note that all ERP null effects have JZS Bayes Factors above 1.

**Table S1: P2 Localizer Statistics**

| **Comparison** | ***Beta*** | ***SE*** | ***df*** | ***t-value*** | ***p-value*** | ***95% CI*** |
| --- | --- | --- | --- | --- | --- | --- |
| Cz > Fz | 1.205 | 0.276 | 665 | 4.366 | <0.001* | [0.663, 1.744] |
| Poz > Cz | 9.338 | 0.276 | 665 | 33.873 | <0.001* | [8.798, 9.878] |
| Poz > Fz | 10.541 | 0.276 | 665 | 38.239 | <0.001* | [10.001, 11.082] |

**Table S2: P3 Localizer Statistics**

| **Comparison** | ***Beta*** | ***SE*** | ***df*** | ***t-value*** | ***p-value*** | ***95% CI*** |
| --- | --- | --- | --- | --- | --- | --- |
| Cz > Fz | 2.202 | 0.218 | 665 | 10.084 | <0.001* | [1.774, 2.630] |
| Poz > Cz | 7.923 | 0.218 | 665 | 36.282 | <0.001* | [7.495, 8.351] |
| Poz > Fz | 10.125 | 0.218 | 665 | 46.367 | <0.001* | [9.697, 10.553] |

**Table S3: Reaction Time Statistics**

| **Effect** | ***Beta*** | ***SE*** | ***df*** | ***t-value*** | ***p-value*** | ***95% CI*** |
| --- | --- | --- | --- | --- | --- | --- |
| Race | -0.004 | 0.006 | 28.400 | -0.624 | 0.538 | [-0.016, 0.008] |
| Status | 0.005 | 0.004 | 28.350 | 1.091 | 0.284 | [-0.004, 0.013] |
| Task | 0.021 | 0.007 | 27.920 | 3.006 | 0.006* | [0.007, 0.035] |
| Race × Status | -0.003 | 0.008 | 27.600 | -0.335 | 0.740 | [-0.019, 0.014] |
| Race × Task | -0.002 | 0.011 | 27.390 | -0.136 | 0.893 | [-0.024, 0.021] |
| Status × Task | -0.036 | 0.010 | 26.620 | -3.462 | 0.002* | [-0.057, -0.016] |
| Race × Status × Task | 0.008 | 0.016 | 25880 | 0.509 | 0.611 | [-0.023, 0.040] |

**Table S4: Reaction Time Decomposition of Status x Task Interaction**

| **Effect** | ***Beta*** | ***SE*** | ***df*** | ***t-value*** | ***p-value*** | ***95% CI*** |
| --- | --- | --- | --- | --- | --- | --- |
| High Status, Race Categorization  vs. High Status, Status Categorization | 0.003 | 0.009 | 51 | 0.347 | 0.73 | [-0.014, 0.020] |
| Low Status, Race Categorization  vs. Low Status, Status Categorization | 0.039 | 0.009 | 51 | 4.478 | < 0.001* | [0.022, 0.056] |
| High Status, Race Categorization  vs. Low Status, Race Categorization | -0.013 | 0.007 | 51 | -1.962 | 0.055 | [-0.021, -0.00002] |
| High Status, Status Categorization  vs. Low Status, Status Categorization | 0.023 | 0.007 | 51 | 3.348 | 0.002* | [0.009, 0.036] |

**Table S5: P200 Statistics**

| **Effect** | ***Beta*** | ***SE*** | ***df*** | ***t-value*** | ***p-value*** | ***95% CI*** | ***Bayes Factor JZS Score*** |
| --- | --- | --- | --- | --- | --- | --- | --- |
| Race | 0.306 | 0.190 | 28 | 1.612 | 0.118 | [-0.066, 0.678] | 1.760 |
| Status | 0.149 | 0.224 | 28 | 0.663 | 0.512 | [-0.290, 0.587] | 24.680 |
| Task | 1.047 | 0.294 | 28 | 3.561 | 0.001* | [0.471, 1.624] |  |
| Race × Status | -0.084 | 0.302 | 28 | -0.279 | 0.783 | [-0.676, 0.508] | 4.769 |
| Race × Task | 0.599 | 0.226 | 84 | 2.645 | 0.010* | [0.155, 1.043] |  |
| Status × Task | -0.315 | 0.226 | 84 | -1.393 | 0.167 | [-0.759, 0.128] | 3.496 |
| Race × Status × Task | 0.891 | 0.453 | 84 | 1.967 | 0.053 | [0.003, 1.778] | 1.922 |

**Table S6: P200 Decomposition of Race x Task Interaction**

| **Effect** | ***Beta*** | ***SE*** | ***df*** | ***t-value*** | ***p-value*** | ***95% CI*** |
| --- | --- | --- | --- | --- | --- | --- |
| Black Faces, Race Categorization  vs. Black Faces, Status Categorization | 0.748 | 0.315 | 36.643 | 2.373 | 0.023* | [0.130, 1.366] |
| White Faces, Race Categorization  Vs. White Faces, Status Categorization | 1.347 | 0.315 | 36.643 | 4.274 | <0.001* | [0.729, 1.964] |
| White Faces, Race Categorization  vs. Black Faces, Race Categorization | 0.605 | 0.221 | 49.376 | 2.740 | 0.009* | [0.172, 1.039] |
| White Faces, Status Categorization  vs. Black Faces, Status Categorization | 0.007 | 0.221 | 49.376 | 0.030 | 0.977 | [-0.427, 0.440] |

**Table S7: P300 Statistics**

| **Effect** | ***Beta*** | ***SE*** | ***df*** | ***t-value*** | ***p-value*** | ***95% CI*** | ***Bayes Factor***  ***JZS Score*** |
| --- | --- | --- | --- | --- | --- | --- | --- |
| Race | 0.098 | 0.157 | 28 | 0.622 | 0.539 | [-0.210, 0.406] | 5.891 |
| Status | 0.057 | 0.109 | 28 | 0.524 | 0.604 | [-0.157, 0.271] | 34.072 |
| Task | 0.249 | 0.313 | 28 | 0.794 | 0.434 | [-0.365, 0.862] | 2.076 |
| Race × Status | -0.057 | 0.184 | 28 | -0.310 | 0.759 | [-0.417, 0.303] | 5.237 |
| Race × Task | 0.664 | 0.190 | 28 | 3.495 | 0.002* | [0.292, 1.036] |  |
| Status × Task | -0.207 | 0.204 | 28 | -1.012 | 0.320 | [-0.607, 0.193] | 4.360 |
| Race × Status × Task | 0.470 | 0.307 | 28 | 1.532 | 0.137 | [-0.131, 1.072] | 3.088 |

**Table S8: P300 Decomposition of Race x Task Interaction**

| **Effect** | ***Beta*** | ***SE*** | ***df*** | ***t-value*** | ***p-value*** | ***95% CI*** |
| --- | --- | --- | --- | --- | --- | --- |
| Black Faces,  Race Categorization  vs. Black Faces,  Status Categorization | -0.083 | 0.327 | 33.108 | -0.254 | 0.801 | [-0.725, 0.558] |
| White Faces,  Race Categorization  vs. White Faces,  Status Categorization | 0.581 | 0.327 | 33.108 | 1.774 | 0.085 | [-0.061, 1.222] |
| White Faces,  Race Categorization  vs. Black Faces,  Race Categorization | 0.430 | 0.184 | 46.043 | 2.340 | 0.024* | [0.070, 0.790] |
| White Faces,  Status Categorization  vs. Black Faces,  Status Categorization | -0.234 | 0.184 | 46.043 | -1.275 | 0.209 | [-0.594, 0.126] |

**Table S9: Alpha Band Statistics in Attention/Executive Function Network**

| **Effect** | ***Beta*** | ***SE*** | ***df*** | ***t-value*** | ***p-value*** | ***95% CI*** |
| --- | --- | --- | --- | --- | --- | --- |
| Race | 0.001 | 0.001 | 196 | 1.531 | 0.127 | [-0.0002, 0.002] |
| Status | -0.001 | 0.001 | 196 | -0.975 | 0.331 | [-0.002, 0.001] |
| Task | -0.0002 | 0.001 | 196 | -0.348 | 0.728 | [-0.001, 0.001] |
| Race × Status | 0.002 | 0.001 | 196 | 1.573 | 0.117 | [-0.0004, 0.004] |
| Race × Task | -0.001 | 0.001 | 196 | -0.769 | 0.443 | [-0.003, 0.001] |
| Status × Task | 0.004 | 0.001 | 196 | 3.889 | <0.001* | [0.002, 0.006] |
| Race × Status × Task | 0.0003 | 0.002 | 196 | 0.166 | 0.868 | [-0.004, 0.004] |

**Table S10: Alpha Band Decomposition of Status x Task Interaction in Attention/Executive Function Network**

| **Effect** | ***Beta*** | ***SE*** | ***df*** | ***t-value*** | ***p-value*** | ***95% CI*** |
| --- | --- | --- | --- | --- | --- | --- |
| High Status,  Race Categorization  vs. High Status,  Status Categorization | 0.002 | 0.001 | 196 | 2.504 | 0.013* | [0.0004, 0.003] |
| Low Status,  Race Categorizations  vs. Low Status,  Status Categorizations | -0.002 | 0.001 | 196 | -2.996 | 0.003* | [-0.004, -0.001] |
| High Status,  Race Categorization  vs. Low Status,  Race Categorization | 0.001 | 0.001 | 196 | 2.061 | 0.041* | [0.00007, 0.003] |
| High Status,  Status Categorization  vs. Low Status,  Status Categorization | -0.003 | 0.001 | 196 | -3.439 | <0.001* | [-0.004, -0.001] |

**Table S11: Alpha Band Statistics in Social Cognitive Network**

| **Effect** | ***Beta*** | ***SE*** | ***df*** | ***t-value*** | ***p-value*** | ***95% CI*** |
| --- | --- | --- | --- | --- | --- | --- |
| Race | 0.0003 | 0.001 | 196 | 0.641 | 0.522 | [-0.001, 0.001] |
| Status | 0.001 | 0.001 | 196 | 2.467 | 0.015* | [0.0003, 0.002] |
| Task | 0.0001 | 0.001 | 196 | 0.229 | 0.819 | [-0.001, 0.001] |
| Race × Status | -0.0002 | 0.001 | 196 | -0.172 | 0.863 | [-0.002, 0.002] |
| Race × Task | 0.001 | 0.001 | 196 | 1.273 | 0.205 | [-0.001, 0.003] |
| Status × Task | 0.001 | 0.001 | 196 | 0.622 | 0.535 | [-0.001, 0.003] |
| Race × Status × Task | -0.0003 | 0.002 | 196 | -0.138 | 0.890 | [-0.005, 0.004] |

## **S10 Figures of All Conditions for Reported Effects**

**Figure S4: Reaction Time**


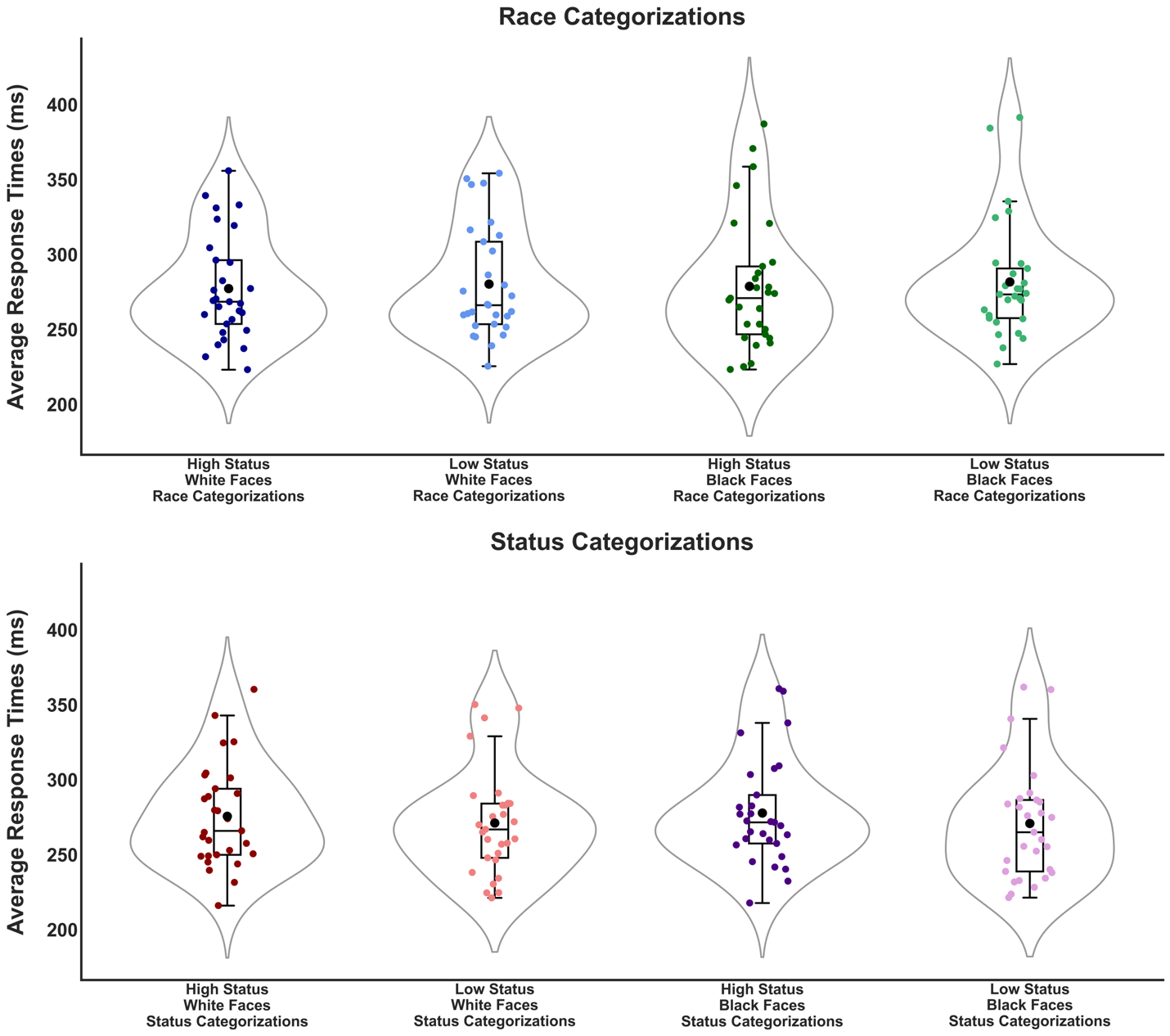


**Figure S4:** *Categorization Speed as a Function of Ascribed Status, Perceived Race, and Categorization Task.* Red and purple dots (bottom panel) represent the status categorization task, and blue and green dots represent the race categorization task (top panel). The y-axis represents reaction times in ms, and the x-axis represents conditions. Lighter colored dots represent low-status faces, and darker colored dots represent high-status faces. The mean and the 95% confidence interval are displayed as a point estimate and horizontal bar (black dot and line, respectively). The boxes indicate the interquartile range (i.e., the 25^th^ and 75^th^ percentiles of these data). A black line and asterisk denote significant simple differences within the interaction.

**Figure S5: P200 Amplitudes**


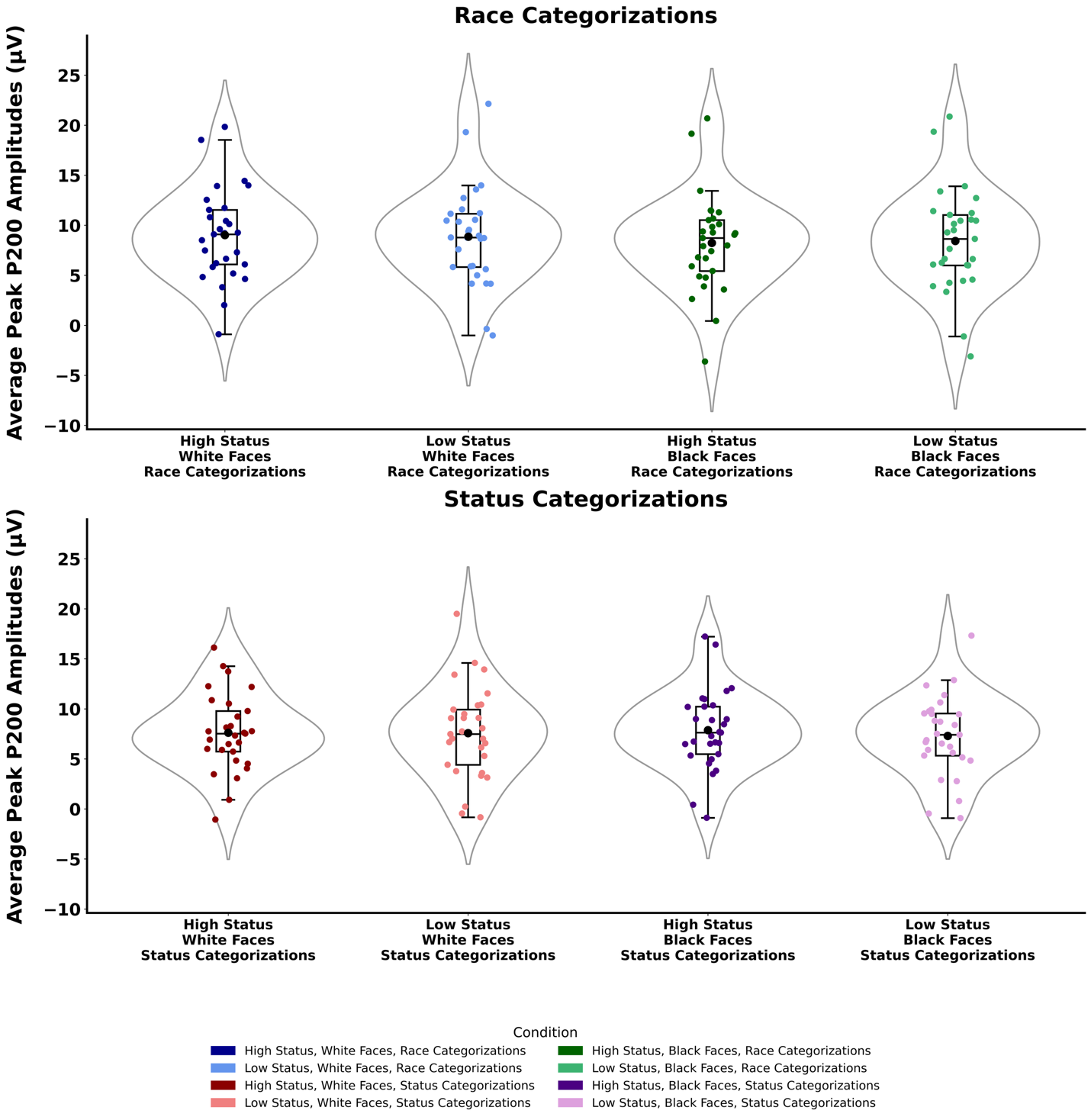


**Figure S5:** *P200 Amplitudes as a Function of Perceived Race and Categorization Task.* Red and purple dots represent the status categorization task (bottom panel), and blue and green dots represent the race categorization task (top panel). The y-axis represents average waveform amplitudes in µV, and the x-axis represents conditions. Lighter colored dots represent low-status faces, and darker colored dots represent high-status faces. The mean and the 95% confidence interval are displayed as a point estimate and horizontal bar (black dot and line, respectively). The boxes indicate the interquartile range (i.e., the 25^th^ and 75^th^ percentiles of these data). A black line and an asterisk denote significant simple differences within the interaction.

**Figure S6: P300 Amplitudes**


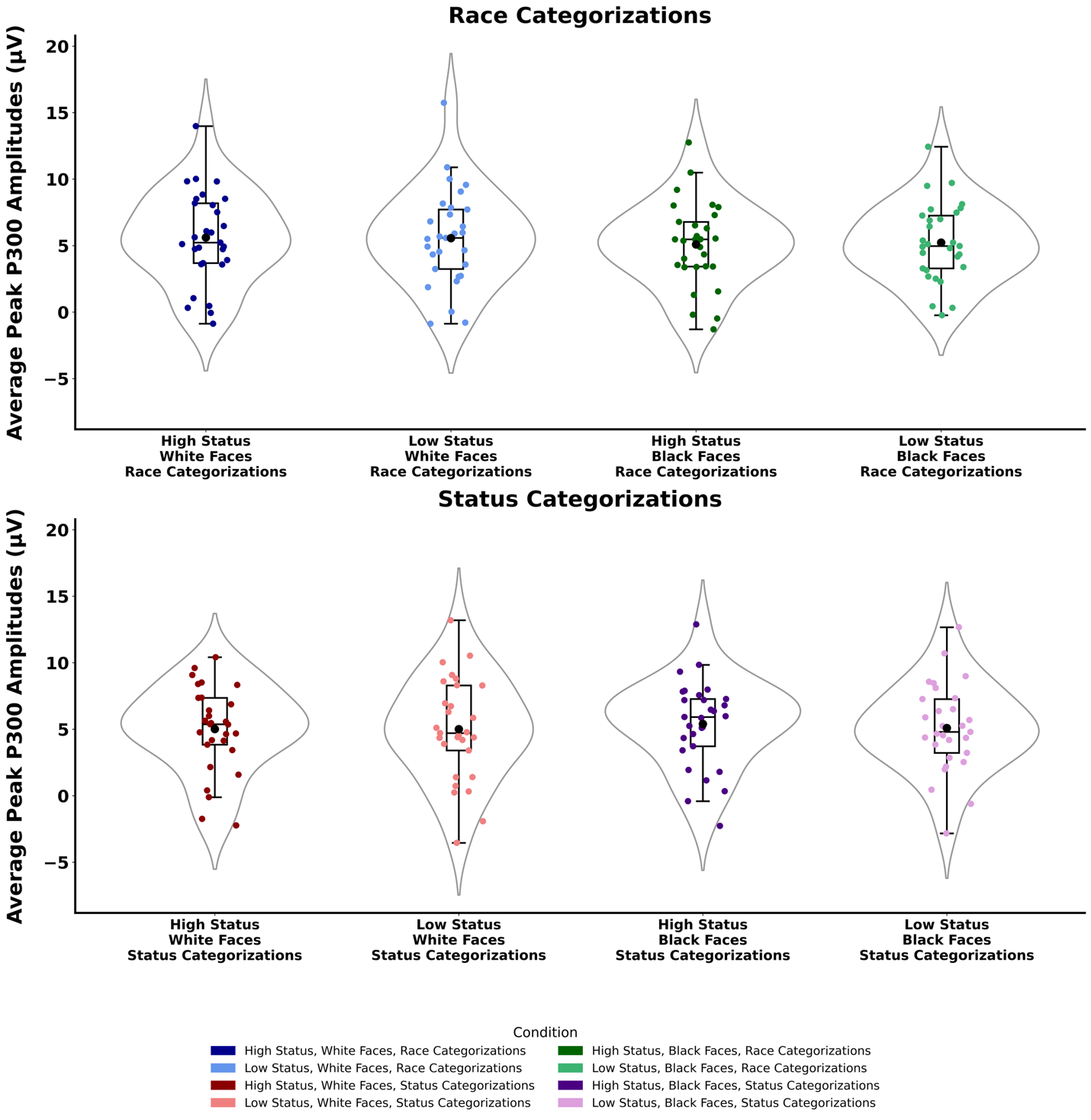
**Figure S6:** *P300 Amplitudes as a Function of Perceived Race and Categorization Task.* Red and purple dots represent the status categorization task (bottom panel), and blue and green dots represent the race categorization task (top panel). The y-axis represents average waveform amplitudes in µV, and the x-axis represents conditions. Lighter colored dots represent low-status faces, and darker colored dots represent high-status faces. The mean and the 95% confidence interval are displayed as a point estimate and horizontal bar (black dot and line, respectively). The boxes indicate the interquartile range (i.e., the 25^th^ and 75^th^ percentiles of these data). A black line and an asterisk denote significant simple differences within the interaction.

**Figure S7: Functional** *α* **Connectivity within the Attention/Executive Function Network**


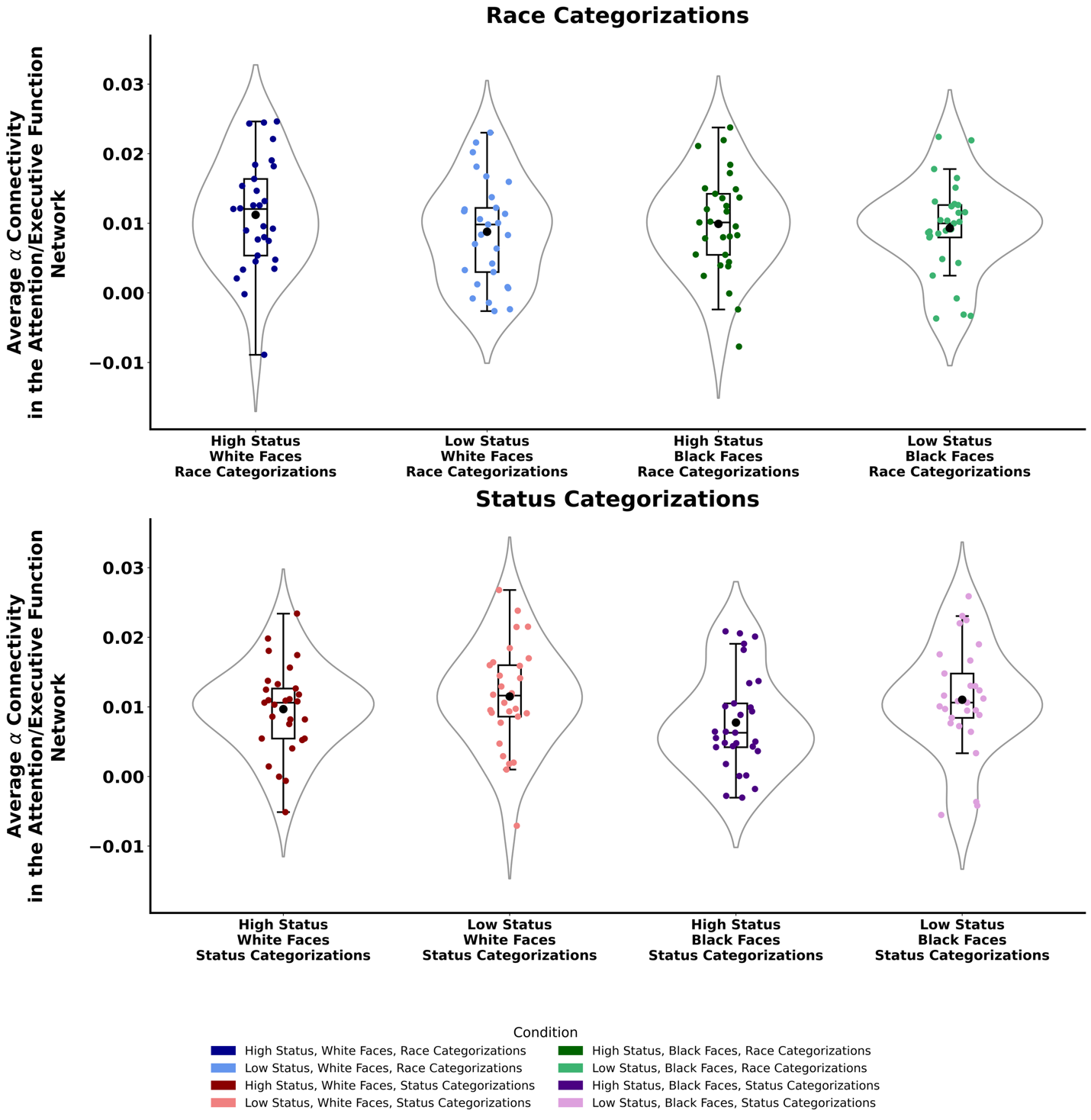


**Figure S7:** *Functional α Connectivity within the Attention/Executive Function Network as a Function of Ascribed Status and Categorization Task.* Red and purple dots represent the status categorization task (bottom panel), and blue and green dots represent the race categorization task (top panel). The y-axis represents average α functional connectivity, and the x-axis represents conditions. Lighter colored dots represent low-status faces, and darker colored dots represent high-status faces. The mean and the 95% confidence interval are displayed as a point estimate and horizontal bar (black dot and line, respectively). The boxes indicate the interquartile range (i.e., the 25^th^ and 75^th^ percentiles of these data). A black line and an asterisk denote significant simple differences within the interaction.

**Figure S8: Functional** *α* **Connectivity within the Social Cognitive/Evaluative Network**


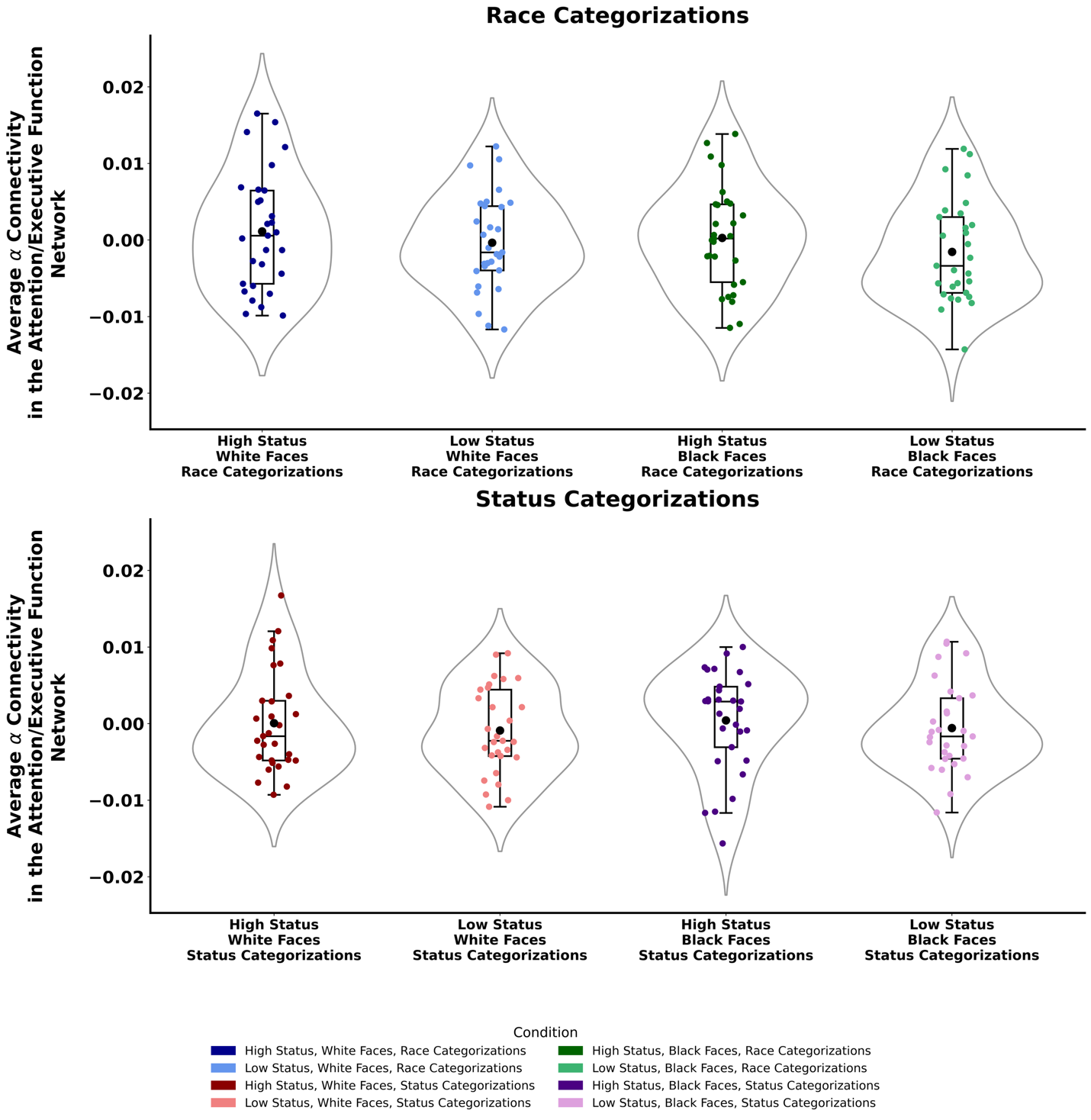


**Figure S8:** *Functional α Connectivity within the Social Cognitive/Evaluative Network as a Function of Ascribed Status and Categorization Task.* Red and purple dots represent the status categorization task (bottom panel), and blue and green dots represent the race categorization task (top panel). The y-axis represents average α functional connectivity, and the x-axis represents conditions. Lighter colored dots represent low-status faces, and darker colored dots represent high-status faces. The mean and the 95% confidence interval are displayed as a point estimate and horizontal bar (black dot and line, respectively). The boxes indicate the interquartile range (i.e., the 25^th^ and 75^th^ percentiles of these data). A black line and an asterisk denote significant simple differences within the interaction.

References

Adler, N. E., Epel, E. S., Castellazzo, G., & Ickovics, J. R. (2000). Relationship of subjective and objective social status with psychological and physiological functioning: Preliminary data in healthy white women. *Health Psychology*, *19*(6), 586–592. https://doi.org/10.1037/0278-6133.19.6.586

Anzures, G., & Mildort, M. (2021). Do perceptual expertise and implicit racial bias predict early face-sensitive ERP responses? *Brain and Cognition*, *147*, 105671.

Bates, D., Kliegl, R., Vasishth, S., & Baayen, H. (2015). Parsimonious Mixed Models. *ArXiv*, *1506*.

Bates, D., Mächler, M., Bolker, B. M., Walker, S. C., Maechler, B., Bolker, B. M., & Walker, S. C. (2015). Fitting linear mixed-effects models using lme4. *Journal of Statistical Software*, *67*(1), 1–48. https://doi.org/10.18637/jss.v067.i01

Bentin, S., & Deouell, L. Y. (2000). Structural encoding and identification in face processing: ERP evidence for separate mechanisms. *Cognitive Neuropsychology*, *17*(1–3), 35–55. https://doi.org/10.1080/026432900380472

Caldara, R., Rossion, B., Bovet, P., & Hauert, C. A. (2004). Event-related potentials and time course of the “other-race” face classification advantage. *Neuroreport*, *15*(5), 905–910.

Chiao, J. Y., Adams, R. B., Tse, P. U., Lowenthal, W. T., Richeson, J. A., & Ambady, N. (2008). Knowing Who’s Boss: fMRI and ERP Investigations of Social Dominance Perception. *Group Processes & Intergroup Relations*, *11*(2), 201–214. https://doi.org/10.1177/1368430207088038

Cloutier, J., Li, T., & Correll, J. (2014). The impact of childhood experience on amygdala response to perceptually familiar Black and White faces. *Journal of Cognitive Neuroscience*, *26*(9), 1992–2004. https://doi.org/10.1162/jocn_a_00605

Cloutier, J., Li, T., Mišić, B., Correll, J., Berman, M. G., Mišic, B., Correll, J., & Berman, M. G. (2017). Brain Network Activity During Face Perception: The Impact of Perceptual Familiarity and Individual Differences in Childhood Experience. *Cerebral Cortex*, *27*, 1–13. https://doi.org/10.1093/cercor/bhw232

Correll, J., Urland, G. R., & Ito, T. A. (2006). Event-related potentials and the decision to shoot: The role of threat perception and cognitive control. *Journal of Experimental Social Psychology*, *42*(1), 120–128. https://doi.org/10.1016/j.jesp.2005.02.006

Dickter, C. L., & Bartholow, B. D. (2007). Racial ingroup and outgroup attention biases revealed by event-related brain potentials. *Social Cognitive and Affective Neuroscience*, *2*(3), 189–198. https://doi.org/10.1093/scan/nsm012

Fazio, R. H., Jackson, J. R., Dunton, B. C., & Williams, C. J. (1995). Variability in automatic activation as an unobtrusive measure of racial attitudes: A bona fide pipeline? *Journal of Personality and Social Psychology*, *69*(6), 1013–1027. https://doi.org/10.1037/0022-3514.69.6.1013

Green, P., & MacLeod, C. J. (2016). SIMR: An R package for power analysis of generalized linear mixed models by simulation. *Methods in Ecology and Evolution*, *7*(4), 493–498.

Ito, T. A., & Bartholow, B. D. (2009). The neural correlates of race. *Trends in Cognitive Sciences*, *13*(12), 524–531. https://doi.org/10.1016/j.tics.2009.10.002

Ito, T. A., & Urland, G. R. (2003). Race and gender on the brain: Electrocortical measures of attention to the race and gender of multiply categorizable individuals. *Journal of Personality and Social Psychology*, *85*(4), 616–626. https://doi.org/10.1037/0022-3514.85.4.616

Ito, T. A., & Urland, G. R. (2005). The influence of processing objectives on the perception of faces: An ERP study of race and gender perception. *Cognitive, Affective & Behavioral Neuroscience*, *5*(1), 21–36. https://doi.org/10.3758/cabn.5.1.21

Kubota, J. T., & Ito, T. A. (2007). Multiple cues in social perception: The time course of processing race and facial expression. *Journal of Experimental Social Psychology*, *43*(5), 738–752. https://doi.org/10.1016/j.jesp.2006.10.023

Kubota, J. T., & Ito, T. A. (2009). You were always on my mind: How event-related potentials inform impression formation research. In T. D. Nelson (Ed.), *Handbook of Prejudice, Stereotyping and Discrimination* (pp. 279–299). Psychology Press.

Kubota, J. T., & Ito, T. A. (2017). Rapid race perception despite individuation and accuracy goals. *Social Neuroscience*, *12*(4), 468–478. https://doi.org/10.1080/17470919.2016.1182585

Kubota, J. T., Peiso, J., Marcum, K., & Cloutier, J. (2017). Intergroup contact throughout the lifespan modulates implicit racial biases across perceivers’ racial group. *PLoS ONE*, *12*(7), e0180440.

Li, T., Cardenas-Iniguez, C., Correll, J., & Cloutier, J. (2016). The impact of motivation on race-based impression formation. *NeuroImage*, *124*, 1–7. https://doi.org/10.1016/j.neuroimage.2015.08.035

Lucas, H. D., Chiao, J. Y., & Paller, K. A. (2011). Why some faces won’t be remembered: Brain potentials illuminate successful versus unsuccessful encoding for same-race and other-race faces. *Frontiers in Human Neuroscience*, *5*.

Ma, D., Correll, J., & Wittenbrink, B. (2015). The Chicago face database: A free stimulus set of faces and norming data. *Behavior Research Methods*, *47*. https://doi.org/10.3758/s13428-014-0532-5

Mattan, B. D., Barth, D. M., Thompson, A., FeldmanHall, O., Cloutier, J., & Kubota, J. T. (2020). Punishing the privileged: Selfish offers from high-status allocators elicit greater punishment from third-party arbitrators. *PLOS ONE*, *15*(5), e0232369. https://doi.org/10.1371/journal.pone.0232369

Mattan, B. D., Kubota, J. T., Li, T., Venezia, S. A., & Cloutier, J. (2019). Implicit evaluative biases toward targets varying in race and socioeconomic status. *Personality and Social Psychology Bulletin*, *45*(10), 1512–1527. https://doi.org/10.1177/0146167219835230

McConahay, J. B. (1986). Modern racism, ambivalence, and the modern racism scale. In J. F. Dovidio & S. L. Gaertner (Eds.), *Prejudice, Discrimination, and Racism* (pp. 91–125). Academic Press.

Michel, C., Rossion, B., Han, J., Chung, C.-S., & Caldara, R. (2006). Holistic processing Is finely tuned for faces of one’s own race. *Psychological Science*, *17*(7), 608–615.

Oakes, J. M., & Rossi, P. H. (2003). The measurement of SES in health research: Current practice and steps toward a new approach. *Social Science and Medicine*, *56*, 769–784. https://doi.org/10.1016/S0277-9536(02)00073-4

Ofan, R. H., Rubin, N., & Amodio, D. M. (2011). Seeing race: N170 responses to race and their relation to automatic racial attitudes and controlled processing. *Journal of Cognitive Neuroscience*, *23*(10), 3153–3161.

Plant, E. A., & Devine, P. G. (1998). Internal and external motivation to respond without prejudice. *Journal of Personality and Social Psychology*, *75*(3), 811–832.

Rouder, J. N., & Morey, R. D. (2012). Default Bayes factors for model selection in regression. *Multivariate Behavioral Research, 47(6*), 877-903.

Rouder, J. N., Speckman, P. L., Sun, D., Morey, R. D., & Iverson, G. (2009). Bayesian t tests for accepting and rejecting the null hypothesis. *Psychonomic Bulletin & Review, 16(2*), 225-237.

Santamaría-García, H., Burgaleta, M., & Sebastián-Gallés, N. (2015). Neuroanatomical Markers of Social Hierarchy Recognition in Humans: A Combined ERP/MRI Study. *The Journal of Neuroscience*, *35*(30), 10843 LP-- 10850. https://doi.org/10.1523/JNEUROSCI.1457-14.2015

Senholzi, K. B., & Ito, T. A. (2013). Structural face encoding: How task affects the N170’s sensitivity to race. *Social Cognitive and Affective Neuroscience*, *8*(8), 937–942. https://doi.org/10.1093/scan/nss091

Stahl, J., Wiese, H., & Schweinberger, S. R. (2008). Expertise and own-race bias in face processing: an event-related potential study. *Neuroreport*, *19*(5), 583--587 10.1097/WNR.0b013e3282f97b4d. http://journals.lww.com/neuroreport/Fulltext/2008/03260/Expertise_and_own_race_bias_in_face_processing__an.15.aspx

Tanaka, J. W., Curran, T., Porterfield, A. L., & Collins, D. (2006). Activation of preexisting and acquired face representations: the N250 event-related potential as an index of face familiarity. *Journal of Cognitive Neuroscience*, *18*(9), 1488–1497.

Walker, P. M., Silvert, L., Hewstone, M., & Nobre, A. C. (2008). Social contact and other-race face processing in the human brain. *Social Cognitive and Affective Neuroscience*, *3*(1), 16–25. https://doi.org/10.1093/scan/nsm035

Westfall, J. (2015). PANGEA: Power ANalysis for GEneral Anova designs. In *PhD Proposal* (Vol. 1). https://doi.org/10.1017/CBO9781107415324.004

Willadsen-Jensen, E. C., & Ito, T. A. (2006). Ambiguity and the timecourse of racial perception. *Social Cognition*, *24*(5), 580–606. https://doi.org/10.1521/soco.2006.24.5.580

Willadsen-Jensen, E. C., & Ito, T. A. (2008). A foot in both worlds: Asian Americans’ perceptions of Asian, White, and racially ambiguous faces. *Group Processes & Intergroup Relations*, *11*(2), 182–200. https://doi.org/10.1177/1368430207088037
